# Brief Web-Based Psychological Intervention for Depressive Symptoms Among Chinese Adolescents: Randomized Controlled Trial

**DOI:** 10.2196/86134

**Published:** 2026-08-07

**Authors:** Pei Liu, Xinwei Wang, Xiaoxia Duan, Mingjing Situ, Yujie Tao, Zhaozhi Yang, Shengnan Zhao, Lei Li, Sixun Li, Yuchu Jiang, Manxue Zhang, Di Jing, Xinyi Yu, Yi Huang

**Affiliations:** 1 Mental Health Center West China Hospital Sichuan University Chengdu China; 2 Schulich School of Medicine and Dentistry Western University London, ON Canada; 3 Laboratory of Child and Adolescent Psychiatry, Mental Health Center West China Hospital Sichuan University Chengdu China

**Keywords:** adolescents, brief web-based psychological intervention, depressive symptoms, randomized controlled trial, RCT, web-based psychoeducation

## Abstract

**Background:**

Adolescents with depressive symptoms are at increased risk of social functional impairment and are more likely to develop major depressive disorder. Digital interventions offer advantages, such as high accessibility, especially for adolescents. However, evidence on the effectiveness of web-based psychological interventions in alleviating depressive symptoms among adolescents remains limited.

**Objective:**

This study aims to develop a brief web-based psychological intervention tailored to the developmental and psychological characteristics of Chinese adolescents and to evaluate its effectiveness and influencing factors.

**Methods:**

In a 2-arm randomized controlled trial, adolescents aged 12 to 18 years with depressive symptoms were recruited from high schools in China. Eligible participants were randomly assigned in a 1:1 ratio to the intervention group (n=212) or the control group (n=224). Participants in the intervention group received a 4-week brief web-based emotional cognitive training program, whereas those in the control group received a web-based psychoeducation program. Nonprofessional helpers provided minimal support through text messages or phone calls. The primary outcome was depressive symptom severity. Secondary outcomes included the depressive symptom remission rate, changes in anxiety symptoms, suicidal ideation, and resilience. Data were collected at baseline (T0), postintervention (T1), and at 1- and 3-month follow-ups (T2 and T3). Statistical analyses included analysis of covariance (ANCOVA), logistic regression analysis, and mediation analysis.

**Results:**

Two-way repeated-measures ANCOVA revealed a significant time effect (*F*_3,1461_=82.515; *P*<.001; η^2^_p_=0.140) on depressive symptom severity, with significantly lower scores at T1, T2, and T3 than at T0. No significant group effect (*F*_1,1461_=1.039; *P*=.31; η^2^_p_=0.001) or time-by-group interaction (*F*_3,1461_=2.424; *P*=.06; η^2^_p_=0.005) for depressive symptom severity was observed. Post hoc exploratory analysis showed that among adolescents with anxiety symptoms, the intervention group exhibited a higher depressive symptom remission rate at T1 than the control group (adjusted odds ratio 1.39, 95% CI 1.05-1.85; *P*=.02; number needed to treat=7). Additionally, the intervention group showed significantly greater improvements in the interpersonal support dimension of resilience at T1 (least squares mean difference=1.79, 95% CI 0.53-3.06; Cohen *d*=0.37, 95% CI 0.11-0.63; *P*=.006; false discovery rate–adjusted *P*=.04). Exploratory mediation analysis identified a significant indirect effect between the intervention and depressive symptom remission via the interpersonal support dimension of resilience at T1 (indirect effect=0.08, 95% CI 0.02-0.14; *P*=.009).

**Conclusions:**

The brief web-based psychological intervention showed no significant difference from web-based psychoeducation in reducing depressive symptoms across the 3-month follow-up period. Post hoc exploratory analyses detected a potential between-group difference in depressive symptom remission among adolescents with comorbid anxiety symptoms, with interpersonal support showing a significant mediating association. These exploratory findings may provide new insights into identifying the target population suitable for brief web-based psychological interventions and inform the development of tailored interventions in the future.

**Trial Registration:**

Chinese Clinical Trial Registry ChiCTR2400090235; https://www.chictr.org.cn/showproj.html?proj=243991

## Introduction

Major depressive disorder (MDD) is a prevalent mental disorder among the general population and has ranked fourth in the global burden of disease in recent years [1]. Evidence suggests that 19% of MDD cases first emerge during adolescence [2]. Given that adolescents are in a critical developmental period characterized by emotional and cognitive growth [3], they are particularly susceptible to various life events that may trigger depressive symptoms and potentially lead to the onset of MDD [4]. The global prevalence of depressive symptoms among adolescents during the COVID-19 pandemic was 25.2% [5]. Adolescents with depressive symptoms may experience impairments in academic and occupational functioning [6,7], and they remain at elevated risk of developing MDD compared with healthy individuals [8-10]. Therefore, psychological interventions targeting adolescents with depressive symptoms represent a critical approach for the early prevention and early intervention of depression.

Cognitive behavioral therapy (CBT) has been widely recommended as a standard intervention for adolescents with depressive symptoms or mild depressive disorders across many countries [11-14]. Although research suggests that CBT is the most evidence-based psychological intervention for depressive symptoms [15], several barriers hinder its widespread implementation, including high intervention costs, substantial time investment from therapists, and limited accessibility [16-18]. School-based CBT interventions can cost up to GBP £43 (US $32) per student [16]. During a full course of CBT treatment, therapists typically spend between 5 and 24 hours providing direct guidance to each participant [17]. Approximately 75% of adolescents with mental health problems have not accessed mental health services [18]. This reluctance to seek help is primarily attributed to a lack of accurate knowledge about mental disorders, high levels of stigma, significant economic burdens, and the inherent inaccessibility of psychotherapy itself [19].

Recent advances in psychotherapy have revealed that specific functional modules within effective interventions demonstrate significant therapeutic value in reducing symptoms [20]. Previous studies have demonstrated that behavioral activation, whether delivered alone or in combination with cognitive restructuring [21,22], as well as psychoeducation [23], mindfulness, relaxation training [24], and peer support modules, can effectively reduce depressive symptoms [25]. Digital therapeutics integrated with internet technology, as well as web-based psychological interventions derived from traditional psychological approaches, have increasingly attracted attention in the field of psychotherapy [26]. Indeed, recent studies have demonstrated that brief web-based psychotherapy provides significant therapeutic effects on depressive symptoms [27-29]. These interventions offer several advantages, including shorter treatment durations, reduced constraints of time and space, high accessibility, and strong scalability. For example, the World Health Organization has released a brief web-based psychological intervention called “Step-by-Step” for adult depressive symptoms [30]. Evidence indicates that “Step-by-Step” can effectively reduce depressive symptoms in both adults and university students, with effects maintained for up to a 3-month follow-up period [31,32].

For adolescent depressive symptoms, studies have demonstrated the efficacy of brief web-based psychological interventions in reducing depressive symptoms. A randomized controlled trial (RCT) involving 128 female adolescents with mild to moderate depressive symptoms evaluated a web-based social cognitive training program. Results showed that the intervention group exhibited significantly lower symptom severity than the blank control group at the 3-month follow-up [33]. A systematic review indicates that most current brief web-based interventions for adolescent depression incorporate psychoeducational components, whereas control groups are typically assigned to waitlist or blank controls [34]. Such designs may introduce placebo effects and overstate the ability to assess the specific therapeutic benefits of the interventions. Therefore, it is essential to implement psychoeducation as an active control condition within an RCT framework to more rigorously evaluate the efficacy of brief CBT-oriented web-based interventions.

This study aims to examine whether a brief web-based psychological intervention can reduce depressive symptoms in Chinese adolescents aged 12 to 18 years and assess the efficacy and sustainability of the intervention effects over a 1- to 3-month follow-up period. Additionally, this study seeks to explore the potential factors influencing the effectiveness of the intervention, such as resilience.

## Methods

### Ethical Considerations

The study protocol was approved by the Ethics Committee on Biomedical Research at West China Hospital, Sichuan University (approval number 2024-1225), and registered in the Chinese Clinical Trial Registry (ChiCTR2400090235). This study was reported in accordance with the CONSORT-EHEALTH checklist (Multimedia Appendix 1). All participants and their parents provided informed consent. Participants received US $15 for completing each of the 1-month and 3-month follow-up assessments. Participants were not compensated for engaging in the intervention. All data were deidentified before analysis to ensure participant confidentiality.

### Study Design

This study used a 2-arm RCT design. From October 2024 to January 2025, eligible adolescents were recruited from 2 junior high schools and 1 senior high school in Chengdu. A total of 457 participants were initially enrolled, and 436 completed randomization and received the assigned interventions. The inclusion criteria were as follows: (1) age between 12 and 18 years; (2) a score of ≥10 on the Patient Health Questionnaire-8 (PHQ-8). Given the sensitivity of items related to “suicide” and “self-harm” in school-based universal screening, the final item of the PHQ-9 was omitted, and the PHQ-8 was used for screening; and (3) all adolescent participants volunteered to take part in the study, and both the participants and their parents provided written informed consent. Outcome assessments were conducted at 4 time points: baseline (T0), postintervention (T1), 1-month follow-up (T2), and 3-month follow-up (T3).

### Procedure

From October 2024 to January 2025, adolescents aged 12 to 18 years with depressive symptoms were recruited from schools to participate in this study. A total of 457 participants were recruited, and 436 underwent randomization. A dedicated researcher stratified the participants by age, gender, school, and depressive symptom severity using a random number generator and subsequently allocated them to either the intervention group or the control group in a 1:1 ratio. Participants in both groups received their assigned interventions and completed outcome assessments at 4 time points. Participants and outcome assessors were not informed of their assigned group or the intervention content delivered to the comparator arm; however, formal verification of blinding status was not conducted during the trial.

### Interventions

Participants in the intervention group received a 4-week, once-weekly web-based psychological intervention through a WeChat mini-program accessible via mobile phone or computer (a screenshot is provided in Figure S1 in Multimedia Appendix 2), whereas those in the control group received web-based psychoeducation with the same frequency and delivered via the identical platform. More details of the intervention modules are provided in Table S1 in Multimedia Appendix 2. Each participant was provided a unique login account and password. Participants were not informed about the content of the other group’s intervention. Four postgraduate students majoring in psychology or psychiatry without medical licenses served as nonprofessional helpers, providing minimal support for platform access and login issues via text messages or phone calls. They did not perform any clinical assessment or therapeutic intervention. The average time commitment per participant was 15 minutes per week for both arms.

### Safety Monitoring

All participants completed the PHQ-8 and the Beck Scale for Suicide Ideation–Chinese Version (BSI-CV) at screening and throughout the intervention period via the online platform. A licensed psychiatrist supervised the risk assessment. An automated alert system was activated when participants scored ≥6 on the BSI-CV to flag suicide risk for psychiatrist review. Based on this review, the psychiatrist determined final study eligibility and decided whether clinical referral was warranted. If a participant explicitly reported worsening depressive symptoms, expressed suicidal ideation, or asked for professional help, the nonprofessional helpers documented the participant’s exact words and escalated the case within 2 hours to the supervising psychiatrist via a secure channel. The psychiatrist then contacted the participant directly, assessed the need for referral, and, if necessary, withdrew the participant from the study to arrange appropriate care.

### Outcome Measurements

#### Overview

Data were collected via the same online platform used for the interventions, with participants completing self-report questionnaires at 4 time points (T0, T1, T2, and T3).

#### PHQ-8

The primary outcome was depressive symptom severity measured by the PHQ-8 [35]. A total score of ≥10 is considered clinically significant and suggests the presence of depressive symptoms [35]. In this study, remission of depressive symptoms was defined as a score of <10 [36]. Score changes were defined as follows: T1–T0 (postintervention minus baseline), T2–T0 (1-month follow-up minus baseline), and T3–T0 (3-month follow-up minus baseline).

#### Generalized Anxiety Disorder-7

Anxiety symptom severity was assessed using the Generalized Anxiety Disorder-7 [37]. A total score of ≥10 is considered indicative of clinically significant anxiety symptoms and may suggest the presence of an anxiety disorder [37].

#### BSI-CV

Suicidal ideation severity was assessed using the BSI-CV [38]. A total score of ≥6 demonstrates the highest classification accuracy for predicting future suicidal behavior [39].

#### Adolescent Resilience Scale

Resilience was evaluated using the Adolescent Resilience Scale [40]. It is a self-report instrument comprising 27 items organized into 5 dimensions: emotion regulation, interpersonal support, family support, positive cognition, and focused targeting. Higher total scores reflect greater resilience and a stronger capacity to cope with stress and adversity.

#### Difficulties in Emotion Regulation Scale

Emotional regulation difficulties were assessed using the Difficulties in Emotion Regulation Scale [41]. It is a self-report measure consisting of 36 items organized into 6 dimensions: lack of emotional awareness, lack of emotional clarity, nonacceptance of emotional responses, impulse control difficulties, difficulties engaging in goal-directed behavior, and limited access to emotion regulation strategies. Higher total scores indicate greater difficulties in emotion regulation.

#### Adolescent Social Support Rating Scale

Social support levels were assessed using the Adolescent Social Support Rating Scale [42]. It is a self-report measure consisting of 17 items organized into 3 dimensions: subjective support, objective support, and support utilization. The average total score reflects the overall level of perceived social support, with higher scores indicating greater availability and more effective use of social support resources.

#### Socioeconomic Status Index

The socioeconomic status (SES) index was calculated using Ren’s method [43], which used principal component analysis to generate standardized scores for parents’ educational level, parents’ occupation type, and family monthly income. Factor loadings were used as weights and multiplied by the corresponding *Z* scores; these weighted values were then summed and divided by the eigenvalue of the first principal component: SES = (β_1_ × *Z*_parents’ education level_ + β_2_ × *Z*_parents’ occupation type_ + β_3_ × *Z*_family monthly income_)/f. In this equation, β_1_, β_2_, and β_3_ represent factor loadings, while f denotes the eigenvalue. The final derived formula in this study is: SES = (0.84 × *Z*_parents’ education level_ + 0.84 × *Z*_parents’ occupation type_ + 0.68 × *Z*_family monthly income_)/0.62.

#### Acceptability and Adverse Events

Regarding the acceptability of the brief web-based psychological intervention program, we analyzed completion rates for both the intervention and control groups at T1, T2, and T3. Additionally, qualitative feedback regarding user experience and suggestions was collected from a subset of students. Technical issues encountered during implementation were reported by the backend developers.

Adverse events, including suicide attempts, self-harm, physical discomfort, and symptom deterioration, were systematically monitored and documented through reports from participants, guardians, or school personnel. Students who reported severe adverse events, such as suicide attempts or self-harm, were followed up and received appropriate psychological or medical support as necessary.

### Statistical Analysis

The sample size was determined using G*Power software (version 3.1). Based on effect size data from a previous RCT, the between-group difference in postintervention depressive symptom scores assessed by the PHQ-9 scale yielded a Hedges *g* of 0.35 [31]. In that trial, the intervention group received a brief web-based psychological intervention, whereas the control group was offered brief web-based psychoeducation and referrals to local mental health services for professional assistance [31]. The statistical power analysis assumed a 2-tailed significance level of α=.05, a desired power of 80% (*β*=0.20), and a 1:1 allocation ratio between groups, yielding a minimum required sample size of 130 participants per group and a total of 260 participants for the study. To account for anticipated participant attrition, we applied a conservative dropout rate of 40%, informed by a pooled attrition rate of 35.5% estimated from a meta-analysis of smartphone-delivered psychological interventions [44]. The adjusted sample size was set at 217 participants per group, resulting in a target total sample size of 434. Eventually, 457 adolescents with depressive symptoms were initially recruited, and 21 participants withdrew before randomization, leaving a final sample of 436 randomized participants.

To assess whether the achieved sample size provided adequate power to detect the effect size observed in this trial, we performed a post hoc sensitivity analysis. Given the final sample of 436 participants (intervention group=212 and control group=224), a 2-tailed independent-samples *t* test with α=.05 and power (1–*β*)=0.80 could detect a minimum standardized mean difference of Cohen *d*=0.27, computed using the R (version 4.5.1; R Foundation for Statistical Computing) *pwr* package (version 1.3-0). This sensitivity analysis indicates that the trial could reliably identify small effects above Cohen *d*=0.27, whereas true treatment effects smaller than this threshold could not be statistically distinguished from 0.

Data were organized and analyzed using R, including the *lme4* (version 1.1-37) and *lmerTest* (version 3.1-3) packages, SPSS (version 25.0; IBM Corp), and Mplus (version 8.3; 2012-2018 Muthen & Muthen) software. Demographic characteristics and baseline outcome measures were compared between the intervention and control groups via 2-tailed independent-samples *t* tests and chi-square tests. For data with multiple time points (scale scores of depressive symptoms or other outcome indicators), 2-way repeated-measures analyses of covariance (ANCOVAs) were used, with time (T0, T1, T2, and T3), group (intervention vs control) effects, and the time-by-group interaction as independent variables. Baseline values of the outcome indicators, age, gender, educational stage, only-child status, family structure, and SES index were included as covariates. For change scores in depressive symptoms or other outcome indicators, 1-way ANCOVAs were used, with group as the independent variable and baseline values of the outcome indicators, age, gender, educational stage, only-child status, family structure, and SES index as covariates. Effect sizes for main effects and interactions were evaluated using η^2^_p_, with 0.01, 0.06, and 0.14 representing small, medium, and large effects, respectively [45]. Between-group effect sizes were calculated via least squares mean differences (LSMDs) and Cohen *d*, along with corresponding 95% CIs. Cohen *d* effect magnitudes were interpreted using conventional thresholds: small (*d*=0.20), medium (*d*=0.50), and large (*d*=0.80) [45]. Logistic regression analyses were conducted to examine the association between group assignment and depressive symptom remission rates at each assessment time point (T1, T2, and T3), adjusting for baseline PHQ-8 score, age, gender, educational stage, only-child status, family structure, and SES index. Number needed to treat (NNT) and odds ratios (ORs) with 95% CIs were derived from the adjusted models [45]. A *P* value of <.05 was considered statistically significant throughout the study. To control for inflated type I error from multiple comparisons, we adopted stratified Benjamini-Hochberg false discovery rate (FDR) correction for indicators in between-group comparisons [46,47]. FDR correction was applied to scores and score changes of anxiety symptoms and suicidal ideation, as well as total and subscale scores and corresponding score changes for resilience, difficulties in emotion regulation, and social support.

To explore potential influencing factors of the intervention and the underlying psychological mechanisms, outcome variables with significant between-group differences were selected as mediators. With group assignment as the independent variable and outcome scores or rates as the dependent variable, we adjusted for age, gender, and baseline levels of each outcome. Mediation analyses were performed using maximum likelihood estimation and 1000 bootstrap resamples to evaluate the significance of indirect effects [48].

## Results

### Participants

**Figure 1:**
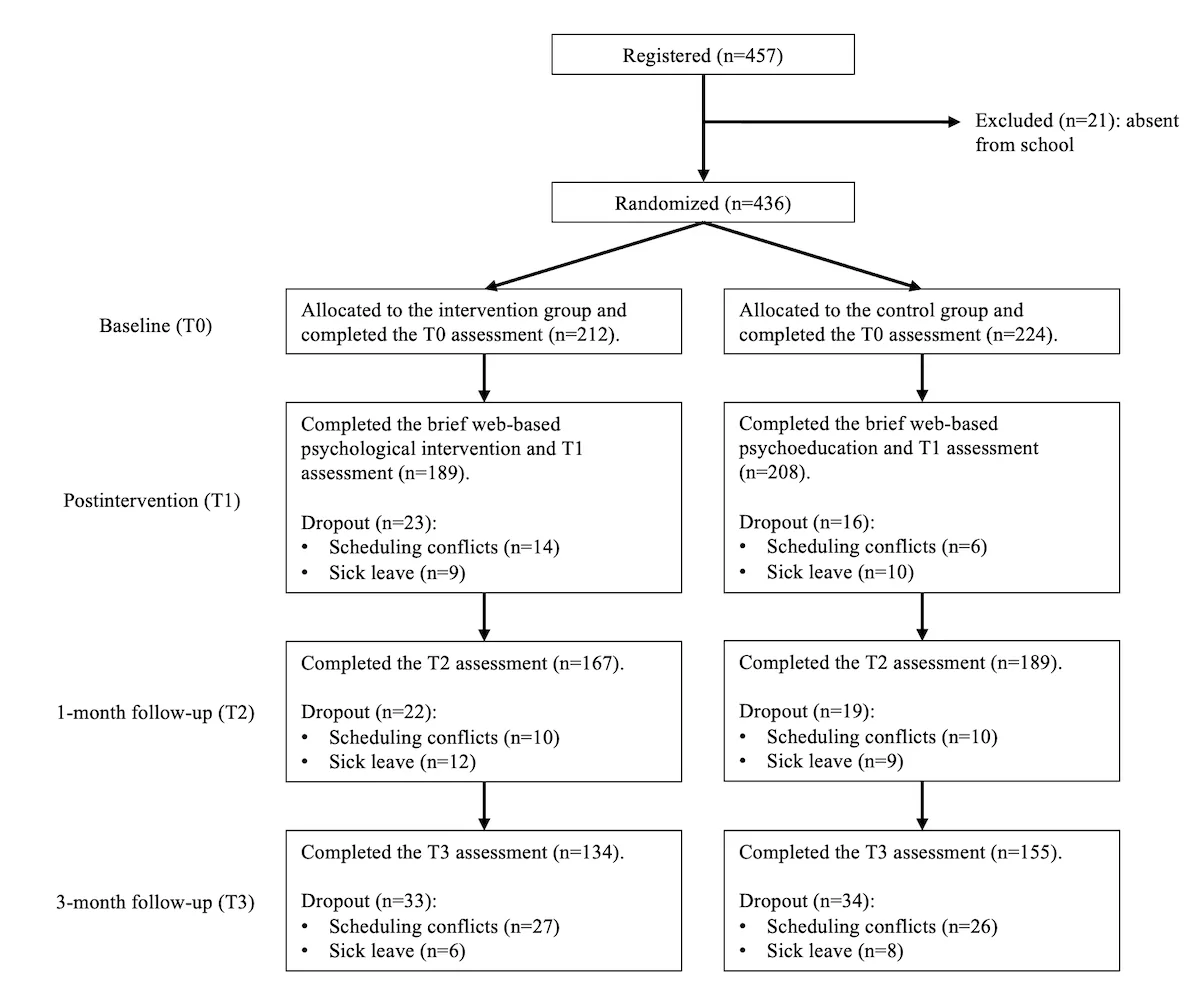
Flow diagram.

From October 2024 to January 2025, adolescents aged 12 to 18 years with depressive symptoms were recruited from schools to participate in this study. A total of 457 participants were recruited, and 436 underwent randomization. Dropout rates differed across time points (Figure 1). For the intervention group (n=212), the rates were 10.9% (23/212) at T1, 10.4% (22/212) at T2, and 15.6% (33/212) at T3. For the control group (n=224), the corresponding rates were 7.1% (16/224) at T1, 8.5% (19/224) at T2, and 15.2% (34/224) at T3. Among the full sample of 436 participants, the mean age was 15.02 (SD 1.49) years. In terms of demographic characteristics, 75.2% (328/436) of participants were female, and 24.8% (108/436) were male; 30.7% (134/436) were junior high school students, while 69.3% (302/436) were high school students. Additionally, 27.3% (119/436) of participants were only children. Regarding family structure, 67% (292/436) of participants came from 2-parent families (residing with both biological or adoptive parents), 9.4% (41/436) from single-parent families (residing with only 1 parent), 6.4% (28/436) from stepfamilies (residing with 1 biological parent and a stepparent), and 17.2% (75/436) did not live with either parent but resided with grandparents or other relatives instead. The SES index ranged from –5.18 to 9.41, with a mean value of 0 (SD 2.99). The mean baseline PHQ-8 score in the full sample was 14.43 (SD 3.02), ranging from 10 to 24. No significant differences were found between the intervention and control groups regarding age, gender, educational stage, only-child status, family structure, SES index, and any outcome indicators (all *P*≥.05; Table 1).

**Table 1:** Demographic characteristics and baseline levels of outcome indicators for the intervention and control groups.

Characters and baseline data			Intervention group (n=212)		Control group (n=224)		*t* test (*df*)/chi-square (*df*)			*P* value	
Age (years), mean (SD)			15.07 (1.43)		14.98 (1.56)		0.617 (434)^a^			.54	
Gender, n (%)								0.599 (1)^b^			.44
	Male	56 (26.4)		52 (23.2)							
	Female	156 (73.6)		172 (76.8)							
Educational stage, n (%)								0.200 (1)^b^			.65
	Junior high school	63 (29.7)		71 (31.7)							
	Senior high school	149 (70.3)		153 (68.3)							
Only child status, n (%)								2.860 (1)^b^			.09
	Only child	50 (23.6)		69 (30.8)							
	Non-only child	162 (76.4)		155 (69.2)							
Family structure, n (%)^c^								3.939 (3)^b^			.27
	2‑parent family	139 (65.6)		153 (68.3)							
	Single‑parent family	16 (7.5)		25 (11.2)							
	Stepfamily	17 (8)		11 (4.9)							
	No parent present	40 (18.9)		35 (15.6)							
Socioeconomic status index, mean (SD)			–0.09 (3.02)		0.08 (2.97)		–0.600 (434)^a^			.55	
Depressive symptom severity, mean (SD)			14.62 (3.01)		14.26 (3.04)		1.240 (434)^a^			.22	
Anxiety symptom severity, mean (SD)			11.43 (4.89)		11.35 (5.21)		0.168 (434)^a^			.87	
Presence of anxiety symptoms^d^, n (%)								0.003 (1)^b^			.96
	With anxiety symptoms	134 (63.2)		141 (62.9)							
	Without anxiety symptoms	78 (36.8)		83 (37.1)							
Suicidal ideation severity, mean (SD)			13.48 (7.63)		12.98 (8.66)		0.633 (431.81)^a^			.53	
Resilience, mean (SD)			72.30 (15.13)		73.77 (17.56)		–0.940 (434)^a^			.35	
	Emotion regulation	13.95 (4.56)		14.23 (5.41)		–0.595 (426.99)^a^			.55		
	Interpersonal support	15.11 (4.97)		15.96 (5.36)		–1.709 (434)^a^			.09		
	Family support	16.59 (5.03)		16.52 (5.32)		0.140 (434)^a^			.09		
	Positive cognition	12.69 (3.41)		12.76 (3.65)		–0.204 (434)^a^			.84		
	Focused targeting	13.96 (4.37)		14.30 (4.41)		–0.814 (434)^a^			.42		
Difficulties in emotion regulation, mean (SD)			113.80 (18.40)		114.35 (20.89)		–0.292 (434)^a^			.77	
	Lack of emotional awareness	16.33 (4.77)		16.00 (4.69)		0.739 (434)^a^			.46		
	Lack of emotional clarity	15.37 (3.29)		15.43 (3.81)		–0.164 (430.53)^a^			.87		
	Nonacceptance of emotional responses	18.98 (5.32)		19.26 (5.51)		–0.544 (434)^a^			.59		
	Impulse control difficulties	19.09 (4.96)		19.62 (5.38)		–1.052 (434)^a^			.29		
	Difficulties engaging in goal-directed behavior	17.54 (4.17)		17.77 (4.49)		–0.542 (434)^a^			.59		
	Limited access to emotion regulation strategies	26.47 (6.37)		26.27 (7.06)		0.309 (434)^a^			.76		
Social support, mean (SD)			3.04 (0.93)		3.17 (1.02)		–1.352 (433.27)^a^			.18	
	Subjective support	3.01 (1.12)		3.12 (1.13)		–1.030 (434)^a^			.30		
	Objective support	3.25 (1.03)		3.34 (1.07)		–0.842 (434)^a^			.40		
	Support utilization	2.86 (1.06)		3.04 (1.20)		–1.660 (432.06)^a^			.10		

^a^Indicates 2-tailed independent-samples *t* test.

^b^Indicates chi-square test.

^c^Family structure was classified into 4 types. 2-parent family: residing with both biological or adoptive parents; single-parent family: residing with only 1 parent; stepfamily: residing with 1 biological parent and a stepparent; no parent present: not living with either parent but staying with grandparents or other relatives.

^d^The criteria for determining the presence of anxiety symptoms were as follows: a total score of ≥10 on the Generalized Anxiety Disorder-7 indicates the presence of anxiety symptoms, whereas a score of <10 indicates the absence of anxiety symptoms.

### Main Results

For all participants, Table 2 presents the ANCOVA-adjusted estimated means and standard errors of each continuous outcome indicator at each time point for the intervention and control groups. The primary outcome (depressive symptom severity, measured by the PHQ-8 score) demonstrated a significant time effect through 2-way repeated-measures ANCOVA (*F*_3,1461_=82.515; *P*<.001; η^2^_p_=0.140), but the group effect (*F*_1,1461_=1.039; *P*=.31; η^2^_p_=0.001) and time-by-group interaction (*F*_3,1461_=2.424; *P*=.06; η^2^_p_=0.005) were not significant. Post hoc comparisons revealed that depressive symptom severity at T1, T2, and T3 was significantly lower than that at T0 in both the intervention and control groups (T1–T0: Cohen *d*=–0.91, 95% CI –1.05 to –0.77; T2–T0: Cohen *d*=–0.90, 95% CI –1.05 to –0.76; T3–T0: Cohen *d*=–0.89, 95% CI –1.05 to –0.74). Significant time effects were also observed for almost all secondary outcomes (*P*_s_<.05; Table S2 in Multimedia Appendix 2). Following FDR correction for multiple testing, none of the secondary outcomes exhibited a statistically significant group effect or time-by-group interaction (all FDR-adjusted *P*≥.05; Table S2 in Multimedia Appendix 2).

**Table 2:** Estimated means and standard errors for continuous outcome indicators at each time point for the intervention and control groups (adjusted for baseline covariates using analysis of covariance).

Outcome indicator and group^a^				Baseline, mean (SE)		Posttest, mean (SE)		1-month follow-up, mean (SE)		3-month follow-up, mean (SE)
Depressive symptom severity										
	Intervention			14.77 (0.43)		9.54 (0.45)		10.04 (0.48)		10.73 (0.53)
	Control			14.65 (0.42)		10.25 (0.44)		9.79 (0.45)		9.23 (0.50)
Anxiety symptom severity										
	Intervention			11.98 (0.38)		9.11 (0.40)		9.16 (0.42)		8.96 (0.47)
	Control			11.91 (0.38)		9.36 (0.39)		8.57 (0.40)		8.26 (0.44)
Suicidal ideation severity										
	Intervention			13.58 (0.52)		10.88 (0.55)		10.09 (0.58)		9.04 (0.64)
	Control			13.41 (0.51)		10.53 (0.53)		9.34 (0.55)		9.22 (0.60)
Resilience dimension										
	Resilience									
		Intervention	72.00 (0.89)		78.60 (0.94)		79.30 (0.99)		78.70 (1.09)	
		Control	72.50 (0.88)		77.00 (0.91)		77.40 (0.94)		77.30 (1.03)	
	Emotion regulation									
		Intervention	13.60 (0.34)		16.90 (0.37)		16.80 (0.38)		17.00 (0.42)	
		Control	13.70 (0.34)		15.90 (0.35)		16.00 (0.37)		16.70 (0.40)	
	Interpersonal support									
		Intervention	15.10 (0.30)		17.40 (0.32)		17.50 (0.34)		12.50 (0.37)	
		Control	15.50 (0.30)		16.50 (0.31)		16.90 (0.32)		11.70 (0.35)	
	Family support									
		Intervention	16.30 (0.32)		17.00 (0.34)		17.20 (0.35)		16.80 (0.39)	
		Control	16.30 (0.31)		17.20 (0.32)		17.20 (0.34)		16.70 (0.37)	
	Positive cognition									
		Intervention	12.80 (0.28)		12.50 (0.30)		12.60 (0.31)		17.70 (0.35)	
		Control	12.80 (0.28)		12.50 (0.29)		12.60 (0.30)		17.90 (0.33)	
	Focused targeting									
		Intervention	14.20 (0.34)		14.70 (0.36)		15.00 (0.38)		14.50 (0.42)	
		Control	14.30 (0.34)		15.00 (0.35)		14.70 (0.36)		14.30 (0.39)	
Emotion regulation dimension										
	Difficulties in emotion regulation									
		Intervention	115.00 (1.34)		113.00 (1.42)		108.00 (1.49)		110.00 (1.65)	
		Control	115.00 (1.32)		113.00 (1.37)		111.00 (1.42)		110.00 (1.56)	
	Lack of emotional awareness									
		Intervention	16.00 (0.40)		16.50 (0.43)		17.30 (0.45)		17.00 (0.50)	
		Control	15.80 (0.40)		16.60 (0.41)		16.80 (0.43)		16.80 (0.47)	
	Lack of emotional clarity									
		Intervention	15.50 (0.22)		15.60 (0.23)		14.90 (0.24)		15.30 (0.27)	
		Control	15.50 (0.22)		15.70 (0.22)		14.90 (0.23)		15.30 (0.26)	
	Nonacceptance of emotional responses									
		Intervention	19.40 (0.45)		19.30 (0.48)		17.80 (0.50)		18.00 (0.55)	
		Control	19.50 (0.44)		19.30 (0.46)		18.60 (0.48)		18.10 (0.52)	
	Impulse control difficulties									
		Intervention	19.50 (0.35)		19.20 (0.38)		18.20 (0.39)		18.40 (0.44)	
		Control	19.70 (0.35)		19.40 (0.36)		18.50 (0.38)		18.40 (0.41)	
	Difficulties engaging in goal-directed behavior									
		Intervention	17.80 (0.29)		16.20 (0.31)		15.60 (0.32)		16.00 (0.35)	
		Control	17.90 (0.28)		16.40 (0.29)		16.40 (0.31)		16.20 (0.33)	
	Limited access to emotion regulation strategies									
		Intervention	26.90 (0.48)		26.00 (0.51)		24.40 (0.53)		24.80 (0.59)	
		Control	26.90 (0.47)		26.10 (0.49)		25.50 (0.51)		25.50 (0.56)	
Social support dimension										
	Social support									
		Intervention	3.07 (0.06)		3.12 (0.07)		3.25 (0.07)		3.32 (0.08)	
		Control	3.11 (0.06)		3.08 (0.07)		3.05 (0.07)		3.22 (0.07)	
	Subjective support									
		Intervention	3.02 (0.07)		3.13 (0.08)		3.24 (0.08)		3.28 (0.09)	
		Control	3.07 (0.07)		3.09 (0.07)		3.05 (0.08)		3.25 (0.08)	
	Objective support									
		Intervention	3.27 (0.07)		3.15 (0.07)		3.33 (0.08)		3.35 (0.08)	
		Control	3.31 (0.07)		3.20 (0.07)		3.17 (0.07)		3.30 (0.08)	
	Support utilization									
		Intervention	2.89 (0.07)		3.06 (0.08)		3.17 (0.08)		3.29 (0.09)	
		Control	2.97 (0.07)		2.97 (0.07)		2.93 (0.08)		3.12 (0.08)	

^a^At baseline, postintervention, 1-month follow-up, and 3-month follow-up, the intervention group included 212, 189, 167, and 134 participants, respectively, while the control group included 224, 208, 189, and 155 participants, respectively.

Furthermore, a significant between-group difference was observed in the change score for the interpersonal support dimension of resilience from T0 to T1 (intervention group: mean change 2.03, SD 0.53; control group: mean change 0.64, SD 0.52; LSMD=1.39, 95% CI 0.42-2.35; Cohen *d*=0.29, 95% CI 0.09-0.49; *P*=.005; FDR-adjusted *P*=.03) through 1-way ANCOVA (Table S3 in Multimedia Appendix 2). Moreover, there was no significant difference in the depressive symptom remission rate between the groups at any time point through logistic regression analysis (Table S4 in Multimedia Appendix 2).

In terms of acceptability, completion rates for both the intervention and control groups across all time points are presented in Table 3. Chi-square tests revealed no significant between-group differences at any assessment point (all *P*≥.05). Furthermore, some participants reported that the visual and textual content was engaging. However, participants reported difficulty finding a quiet environment for audio playback, and the password reset process was troublesome when they forgot their credentials. The backend developers resolved the network latency issue during intervention delivery and shortened the password reset procedure to improve user experience.

No severe adverse events, such as suicide attempts or self-harm behaviors, were reported during the intervention or follow-up period. In this trial, 3 participants withdrew because of self-reported worsening depressive symptoms that required clinical referral. No physical discomfort related to program use was reported.

**Table 3:** Comparison of completion rates between the intervention and control groups across different time points.

Time points	Intervention group (n=212), n (%)	Control group (n=224), n (%)	Chi-square (*df*)	*P* value
Postintervention	189 (89.2)	208 (92.9)	1.837 (1)	.18
1-month follow-up	167 (78.8)	189 (84.4)	2.281 (1)	.13
3-month follow-up	134 (63.2)	155 (69.2)	1.748 (1)	.19

### Post Hoc Exploratory Analysis Results

Adolescents with depressive symptoms may exhibit substantial individual heterogeneity, and intervention effects may differ between those with and without anxiety symptoms. Therefore, participants were divided into 2 subgroups (with anxiety symptoms vs without anxiety symptoms), and the statistical analyses were performed separately within each subgroup. Results are reported in the subsequent section. Demographic characteristics and baseline scale score differences between the intervention and control groups within each subgroup are presented in Table S5 in Multimedia Appendix 2.

**Figure 2:**
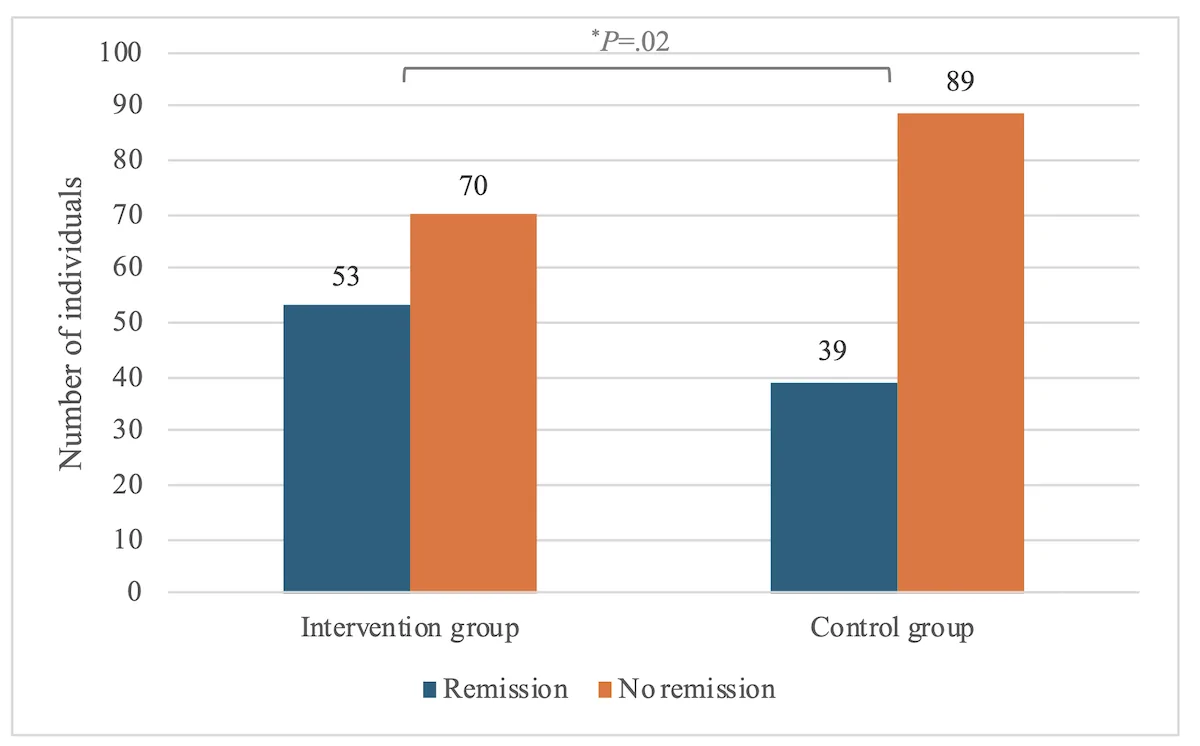
The number of individuals with remission and no remission of depressive symptoms in the intervention and control groups among adolescents with anxiety symptoms at postintervention. Remission of depressive symptoms is defined as a Patient Health Questionnaire-8 (PHQ-8) score of <10 (below the clinical cutoff point). *P<.05; **P<.01; ***P<.001.

Among adolescents with anxiety symptoms, logistic regression analyses indicated a significantly higher depressive symptom remission rate at T1 in the intervention group relative to the control group (intervention: 53/123, 43.1%; control: 39/128, 30.5%; adjusted OR=1.39, 95% CI 1.05-1.85; *P*=.02; NNT=7; Figure 2 and Table S4 in Multimedia Appendix 2). No significant between-group differences were detected in raw scores or changes in depressive symptom severity at any time point in this subgroup (all *P*≥.05; Tables S6-S8 in Multimedia Appendix 2). One-way ANCOVA further showed that the intervention group exhibited substantially greater improvements in the interpersonal support dimension of resilience from T0 to T1 (intervention group: mean change 2.75, SD 0.69; control group: mean change 0.96, SD 0.64; LSMD=1.79, 95% CI 0.53-3.06; Cohen *d*=0.37, 95% CI 0.11-0.63; *P*=.006; FDR-adjusted *P*=.04; Table S8 in Multimedia Appendix 2).

**Figure 3:**
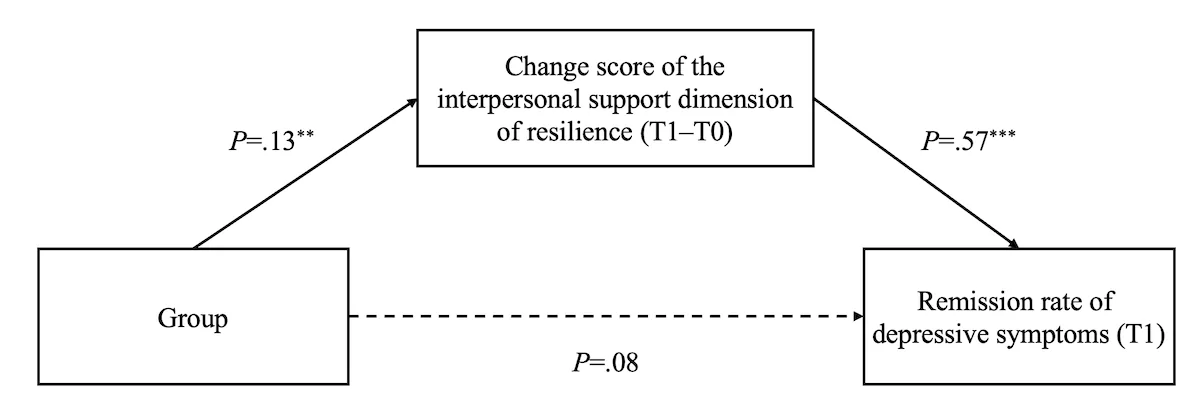
The mediating mechanism on remission of depressive symptoms. The group is a binary categorical variable, with the “intervention group” coded as 1 and the “control group” coded as 0. T0 and T1 refer to the baseline and postintervention time points, respectively. *P<.05; **P<.01; ***P<.001.

Building on these findings observed among adolescents with anxiety symptoms, we constructed an exploratory mediation model. The analysis revealed a statistical association between group assignment and changes in the interpersonal support dimension of resilience from T0 to T1 (*β*=0.13, 95% CI 0.03-0.23; *P*=.008). In turn, changes in interpersonal support were associated with depressive symptom remission at T1 (*β*=0.57, 95% CI 0.41-0.69; *P*<.001). A significant indirect effect was observed across the sequence: group → changes in interpersonal support from T0 to T1 → depressive symptom remission at T1 (*β*=0.08, 95% CI 0.02-0.14; *P*=.009; Figure 3). The proportion mediated was 49.8% (95% CI 15.5%-169.5%). The upper bound exceeding 100% reflects sampling variability and the instability of ratio-based mediation metrics when the total effect is not statistically significant. We therefore focus on the indirect effect as the primary evidence for mediation.

Among adolescents without anxiety symptoms, no significant between-group differences were found in depressive symptom severity, any other outcome measures, or depressive symptom remission rates (all *P*≥.05; all FDR-adjusted *P*≥.05; Tables S4, S6, S9, and S10 in Multimedia Appendix 2).

## Discussion

### Principal Findings

This study reveals that both the brief web-based psychological intervention and web-based psychoeducation are associated with short-term reductions in adolescent depressive symptoms, with improvements sustained from postintervention to the 1- to 3-month follow-up period. No significant between-group differences were observed in any outcome measures. However, unplanned, hypothesis-generating post hoc exploratory analyses among adolescents with anxiety symptoms indicated that the intervention group exhibited a higher depressive symptom remission rate and greater improvement in the interpersonal support dimension of resilience at T1 relative to the control group. Additionally, the association between the intervention and depressive symptom remission was partially explained by improvements in interpersonal support. Given that these findings came from unplanned post hoc analyses, they should be interpreted cautiously and require further validation in rigorous trials.

Both groups exhibited significant reductions in depressive symptom severity up to the 3-month follow-up, which aligns with prior studies of web-based psychological interventions for youth. RCTs have shown that when the control group receives web-based psychoeducation or supportive therapy, both the intervention and control groups exhibit significant decreases in depressive symptom scores, with effects lasting up to the 1- to 3-month follow-up [49,50]. These findings align with the present results, suggesting that web-based psychoeducation alone may relieve depressive symptoms via emotion recognition, regulation training, and social skills modules, which may help adolescents build self-confidence and improve emotional well-being, thereby reducing depressive symptoms. Consistent with several previous trials, we found no group differences in depressive symptom severity when the control group received web-based psychoeducation [51,52]. By contrast, when the control group received treatment as usual or a blank control, the intervention group showed significantly lower depressive symptom scores postintervention or at the 3- to 6-month follow-up [33,53,54]. Notably, significant between-group differences were observed in several subdimensions of resilience and social support. However, these differences did not survive after multiple-comparison correction (Tables S2 and S3 in Multimedia Appendix 2), indicating that they should be interpreted as exploratory rather than confirmatory. Nonetheless, the observed trend toward improved adolescent resilience and social support following web-based psychological intervention aligns with prior evidence [55,56]. This may be attributed to the combined effects of identifying personal strengths within the psychoeducation module of the brief web-based psychological intervention, as well as the skill modules of behavioral activation, cognitive restructuring, peer support, mindfulness, and relaxation training [57-59]. In summary, the brief web-based intervention showed no advantages over web-based psychoeducation in reducing depressive symptoms across all assessed time points. Future work should extend follow-up to 6 to 12 months to evaluate long-term effects and add a blank control group to identify the specific components of psychoeducation that contribute to its effectiveness.

Among adolescents with anxiety symptoms, post hoc analyses revealed a higher postintervention depressive symptom remission rate in the intervention group, whereas no group difference was found in overall depressive symptom changes from baseline to postintervention and the 1- to 3-month follow-ups. Previous studies have reported contradictory findings regarding effects on depressive symptom severity and remission rate. Two RCTs with larger sample sizes (n=160 and n=187) yielded results similar to those of this study, showing significantly higher depressive symptom remission rates in the intervention group but no significant between-group differences in depressive symptom scores [60,61]. In contrast, an RCT with a smaller sample size (n=34) reported the opposite results: significant differences in depressive symptom severity but not in depressive symptom remission rate [50]. In these studies, the control groups received either web-based psychoeducation or treatment as usual, and criteria for depressive symptom remission were both defined as a score less than the clinical cutoff on the depression scales. These findings suggest that larger sample sizes may be more likely to detect significant differences in depressive symptom remission rates. Notably, using a PHQ-8 cutoff of 10 for both enrollment and remission may lead to biased classification due to mean regression. To address this concern, we excluded participants with baseline PHQ-8 scores of 10 to 11 (full sample: 39/436, 8.9%; subgroup with anxiety symptoms: 17/275, 6.2%) and reconducted sensitivity analyses for all significant outcomes. The results remained statistically significant, demonstrating that our main findings are relatively robust (Multimedia Appendix 2).

Both the full sample and the subgroup with anxiety symptoms showed small but statistically significant between-group differences in changes in the interpersonal support dimension of resilience from baseline to postintervention (full sample: Cohen *d*=0.29, 95% CI 0.09-0.49; subgroup with anxiety symptoms: Cohen *d*=0.37, 95% CI 0.11-0.63). Despite statistical significance, the small effect size suggests limited clinical practicality, as such minor changes are unlikely to improve daily functioning or clinical outcomes. This exploratory result can only serve as a preliminary reference for intervention design, rather than evidence of robust clinical efficacy. Mediation analyses further revealed that improved interpersonal support partially mediated the association between the intervention and depressive symptom remission in the anxiety subgroup. As a core component of adolescent resilience, interpersonal support reflects the ability to seek comfort and express emotions through social connections [40]. The peer support module in our intervention may have strengthened this capacity, helping adolescents access more psychological resources, cope better with stress, and recover from distress [62]. A prior RCT involving 91 adolescents with MDD found that higher baseline levels of resilience predicted lower depressive symptom levels after receiving CBT [63]. These findings indicate that the interpersonal support dimension of resilience not only serves as a protective factor against depressive symptoms before intervention but may also predict better postintervention outcomes. Therefore, future web-based psychological interventions should prioritize resilience-building strategies. Unfortunately, these benefits were not maintained at the 1- to 3-month follow-ups, highlighting the need for longer follow-up assessments to evaluate long-term efficacy.

Compared with adolescents with anxiety symptoms, adolescents without anxiety symptoms showed no significant between-group differences in any outcome measures, which is consistent with previous research. In an RCT involving 151 adults with MDD, participants in the intervention group received traditional face-to-face cognitive therapy, whereas those in the control group received interpersonal psychotherapy. The study found that anxiety symptoms positively predicted greater reductions in depressive symptoms in the cognitive therapy group at postintervention [64]. Another RCT including 268 adults with persistent depressive disorder found that participants receiving face-to-face CBT exhibited significantly lower depressive symptom scores and a higher remission rate among those with comorbid anxiety disorders than among those without comorbid anxiety disorders [65]. The co-occurrence of depressive and anxiety symptoms during adolescence is relatively common [66]. Another study showed that among youth with anxiety disorders receiving self-guided CBT, the subgroup exhibiting severe depressive and anxiety symptoms, poor sleep quality, and high levels of negative emotion experienced greater alleviation of both depressive and anxiety symptom severity following intervention [67]. Multiple large-scale epidemiological studies in China have reported a comorbidity rate of depression and anxiety of 11.3% among adolescents [68]. Compared with individuals presenting with either depressive or anxiety symptoms alone, those with both depressive and anxiety symptoms may experience more severe impairments in social functioning, underscoring the need for targeted interventions in this population [69]. The observed intervention effects exhibited considerable heterogeneity within the adolescent population. Future research should aim to refine intervention protocols and develop more tailored psychological intervention strategies to enhance treatment outcomes for specific subgroups.

In terms of intervention acceptability, the overall completion rate reached 89.2% at postintervention, comparable to the average rate of 88.3% reported in similar web-based psychological interventions [70]. We summarized participants’ reasons for withdrawal: some dropped out because of academic conflicts, for which we coordinated with teachers to adjust schedules and facilitate participation; others withdrew because of technical failures, which were resolved promptly by the technical team. Future improvements should optimize intervention content, technical delivery, and implementation based on withdrawal patterns. No severe adverse events, such as suicide attempts or self-harm behaviors, occurred during the trial.

### Limitations

First, this study lacked a no-intervention blank control group. Both the web-based psychological intervention and psychoeducation reduced adolescents’ depressive symptoms, yet no significant differences were found between the 2 interventions. Adding a blank control group in future research will help identify the effective components of psychoeducation. Second, no formal clinical diagnostic assessments were conducted, so the intervention efficacy among adolescents with diagnosed depression remains unknown. Standard diagnostic procedures are needed in future studies to stratify participants accurately and design targeted interventions. Third, data on family-level factors, including siblings’ mental health and parental physical or psychiatric illness, were not collected. These factors influence the development of adolescent depression and intervention responses [71-74]. Systematic assessment of family characteristics is warranted to explore how family dynamics interact with online interventions. Fourth, rigorous allocation concealment was implemented during randomization to prevent predictable group assignment prior to enrollment. Nevertheless, we did not conduct formal checks to confirm whether participants and outcome assessors remained unaware of group allocation after randomization, which may introduce performance bias and detection bias. Future trials should strictly enforce and verify blinding protocols. Fifth, a post hoc sensitivity analysis revealed that, given the final sample size (n=436), the study could detect only between-group differences of Cohen *d*≥0.27. All observed effect sizes for the primary outcome were below this threshold. Therefore, the study was underpowered to detect the true effect size achievable when comparing 2 active interventions, which may partly explain the nonsignificant group effects and interactions. Future trials should consider larger sample sizes or alternative designs to detect small but potentially meaningful effects. Finally, follow-up was limited to 1 and 3 months after the intervention. Extended follow-up of 6 to 12 months is necessary to assess the long-term outcomes of this intervention.

### Conclusions

In this RCT comparing a brief web‑based psychological intervention with an active web‑based psychoeducation control among adolescents with depressive symptoms, the intervention showed no superior effects relative to the active control in reducing depressive symptom severity at any follow-up time point. However, in exploratory subgroup analyses among adolescents with comorbid anxiety symptoms, the intervention suggested a possible improvement in depressive symptom remission at postintervention, which was partially mediated by improvements in the interpersonal support dimension of resilience at that time point. These findings suggest that future studies should strengthen the peer support components, optimize intervention content to deliver targeted psychological support tailored to adolescents with and without anxiety symptoms, and extend the follow-up period to assess the long-term effects of the intervention on depressive symptom reduction.

## Appendix

Multimedia Appendix 1 CONSORT-eHEALTH checklist (V 1.6.1).

Multimedia Appendix 2 Supplementary materials: detailed intervention information and complete results encompassing secondary outcome measures and post hoc exploratory analyses.

## Abbreviations

ANCOVA: analysis of covariance
BSI-CV: Beck Scale for Suicide Ideation–Chinese Version
CBT: cognitive behavioral therapy
FDR: false discovery rate
LSMD: least squares mean difference
MDD: major depressive disorder
NNT: number needed to treat
OR: odds ratio
PHQ-8: Patient Health Questionnaire-8
RCT: randomized controlled trial
SES: socioeconomic status

## Data Availability

The datasets generated or analyzed during this study are available from the corresponding author on reasonable request.
