# Supplementary material for: Brief Web-Based Psychological Intervention for Depressive Symptoms Among Chinese Adolescents: Randomized Controlled Trial
Source: JMIR Mhealth Uhealth. 2026 Aug 7;14:e86134. doi: 10.2196/86134 (PMC13498909; doi:10.2196/86134)
Supplement: Supplementary file 2 [file mhealth_v14i1e86134_app2.docx]

# **Supplementary Material**

## **Figure S1.** **Screenshots of the Wechat mini-program platform.**

## **Table S1. Outline of the brief web-based interventions.**

| **Items** | **Brief web-based psychological intervention** | **Web-based psychoeducation** |
| --- | --- | --- |
| **Components** | (1) Behavioral activation is implemented throughout the entire intervention. Each session includes components designed to promote engagement in pleasant activities, initiation of positive behaviors, and overcoming behavioral obstacles. Immediate positive feedback is provided after each exercise in the form of earned points.  (2) Psychoeducation includes identification of personal strengths, emotion recognition and regulation, identification and prevention strategies for depression and anxiety disorders.  (3) Cognitive restructuring includes guided self-talk exercises.  (4) Mindfulness and relaxation training.  (5) Peer support. Interacting with peers within the system enables users to receive emotional support and companionship. | (1) Emotional psychoeducation encompasses general psychological knowledge as well as content related to self-emotional awareness and regulation, including self-awareness, effective communication skills with peers and teachers, and emotion regulation strategies. It does not include specialized skill training targeting depressive or anxiety symptoms. |
| **Frequency** | The intervention consists of 4 sessions, each lasting 20 to 30 minutes, delivered once per week, with completion expected within 4 weeks. | The intervention consists of 4 sessions, each lasting 10 to 15 minutes, delivered once per week, with completion expected within 4 weeks. |
| **Form** | Multimedia content delivery (including images, text, videos, and interactive components, combined with peer support). | |
| **Guidance** | Trained nonprofessional helpers, who lack formal qualifications as psychiatrists or psychotherapists, deliver minimal and structured support, including session reminders, weekly assignment notifications, technical assistance with platform usage, and adverse event reporting through text messages and phone calls. The average time commitment per participant is 15 minutes per week. | |

## **Table S2. Between-group comparisons for scores of outcome indicators at postintervention, 1-month follow-up, and 3-month follow-up assessments.** T0: baseline. T1: postintervention. T2: 1-month follow-up. T3: 3-month follow-up. FDR: false discovery rate correction.

| **Measures** | **Time effect** | | | | **Group effect** | | | | | **Time-by-group interaction** | | | |
| --- | --- | --- | --- | --- | --- | --- | --- | --- | --- | --- | --- | --- | --- |
|  | ***F* (df)** | ***P* value** | **η^2^_p_** | **Post hoc comparisons Cohen *d* (95%CI)** | ***F* (df)** | ***P* value** | **FDR adjusted *P* value** | **η^2^_p_** | **Between-group comparisons (intervention group-control group) Cohen *d* (95%CI)** | ***F* (df)** | ***P* value** | **FDR adjusted *P* value** | **η^2^_p_** |
| **Depressive symptom severity** | *82.515 (3,1461)* | *<.001* | 0.140 | *T1-T0: –0.91 (–1.05 to –0.77)*  *T2-T0: –0.90 (–1.05 to –0.76)*  *T3-T0: –0.89 (–1.05 to –0.74)* | 1.039 (1,1461) | .31 | - | 0.001 | 0.05 (–0.05 to 0.16) | 2.424 (3,1461) | .06 | - | 0.005 |
| **Anxiety symptom severity** | *42.204 (3,1461)* | *<.001* | 0.080 | *T1-T0: –0.57 (–0.71 to –0.44)*  *T2-T0: –0.65 (–0.80 to –0.51)*  *T3-T0: –0.71 (–0.86 to –0.55)* | 1.204 (1,1461) | .27 | .42 | 0.001 | 0.06 (–0.05 to 0.16) | 0.802 (3,1461) | .49 | .83 | 0.002 |
| **Suicidal ideation severity** | *34.646 (3,1461)* | *<.001* | 0.070 | *T1-T0: –0.43 (–0.57 to –0.30)*  *T2-T0: –0.59 (–0.73 to –0.45)*  *T3-T0: –0.68 (–0.83 to –0.53)* | 0.643 (1,1461) | .42 | .42 | <0.001 | 0.04 (–0.06 to 0.15) | 0.293 (3,1461) | .83 | .83 | 0.001 |
| **Resilience** | *28.079 (3,1460)* | *<.001* | 0.050 | *T1-T0: 0.50 (0.37 to 0.64)*  *T2-T0: 0.55 (0.41 to 0.69)*  *T3-T0: 0.52 (0.37 to 0.67)* | 3.621 (1,1460) | .06 | .11 | 0.002 | 0.10 (–0.00 to 0.21) | 1.015 (3,1460) | .38 | .77 | 0.002 |
| Emotion regulation | *46.602 (3,1460)* | *<.001* | 0.090 | *T1-T0: 0.65 (0.51 to 0.79)*  *T2-T0: 0.64 (0.50 to 0.78)*  *T3-T0: 0.75 (0.60 to 0.90)* | 4.622 (1,1460) | .03 | .10 | 0.003 | 0.12 (0.01 to 0.22) | 1.408 (3,1460) | .24 | .72 | 0.003 |
| Interpersonal support | *119.132 (3,1460)* | *<.001* | 0.200 | *T1-T0: 0.43 (0.29 to 0.57)*  *T2-T0: 0.50 (0.36 to 0.64)*  T3-T0: 0.07 (–0.07 to 0.22) | 6.038 (1,1460) | .01 | .08 | 0.004 | 0.13 (0.03 to 0.24) | 2.818 (3,1460) | .04 | .23 | 0.006 |
| Family support | *4.844 (3,1460)* | *0.002* | 0.010 | *T1-T0: 0.22 (0.08 to 0.35)*  *T2-T0: 0.24 (0.10 to 0.38)*  T3-T0: 0.13 (–0.02 to 0.28) | 0.018 (1,1460) | .89 | .92 | <0.001 | –0.01 (–0.11 to 0.10) | 0.115 (3,1460) | .95 | .98 | <0.001 |
| Positive cognition | *163.855 (3,1460)* | *<.001* | 0.250 | T1-T0: –0.09 (–0.22 to 0.05)  T2-T0: –0.05 (–0.19 to 0.09)  *T3-T0: 1.42 (1.26 to 1.58)* | 0.140 (1,1460) | .71 | .92 | <0.001 | –0.02 (–0.12 to 0.08) | 0.058 (3,1460) | .98 | .98 | <0.001 |
| Focused targeting | 2.078 (3,1460) | .10 | 0.004 | *T1-T0: 0.14 (0.00 to 0.28)*  *T2-T0: 0.15 (0.01 to 0.29)*  T3-T0: 0.05 (–0.10 to 0.20) | 0.010 (1,1460) | .92 | .92 | <0.001 | 0.01 (–0.10 to 0.11) | 0.409 (3,1460) | .75 | .98 | 0.001 |
| **Difficulties in emotion regulation** | *9.808 (3,1461)* | *<.001* | 0.020 | T1-T0: –0.12 (–0.26 to 0.02)  *T2-T0: –0.34 (–0.48 to –0.20)*  *T3-T0: –0.31 (–0.46 to –0.16)* | 1.364 (1,1461) | .24 | .47 | 0.001 | –0.06 (–0.17 to 0.04) | 0.326 (3,1461) | .81 | >.99 | 0.001 |
| Lack of emotional awareness | *4.038 (3,1461)* | *.007* | 0.008 | T1-T0: 0.13 (–0.00 to 0.27)  *T2-T0: –0.23 (–0.37 to –0.09)*  *T3-T0: –0.20 (–0.35 to –0.05)* | 0.702 (1,1461) | .40 | .47 | <0.001 | –0.04 (–0.15 to 0.06) | 0.209 (3,1461) | .89 | >.99 | <0.001 |
| Lack of emotional clarity | *4.759 (3,1461)* | *.003* | 0.010 | T1-T0: 0.07 (–0.06 to 0.21)  *T2-T0: –0.19 (–0.33 to –0.05)*  T3-T0: –0.07 (–0.22 to 0.08) | 0.020 (1,1461) | .89 | .89 | <0.001 | –0.01 (–0.11 to 0.10) | 0.017 (3,1461) | >.99 | >.99 | <0.001 |
| Nonacceptance of emotional responses | *6.072 (3,1461)* | *<.001* | 0.010 | T1-T0: –0.03 (–0.16 to 0.11)  *T2-T0: –0.23 (–0.37 to –0.09)*  *T3-T0: –0.24 (–0.39 to –0.09)* | 0.784 (1,1461) | .38 | .47 | 0.001 | –0.05 (–0.15 to 0.06) | 0.373 (3,1461) | .77 | >.99 | 0.001 |
| Impulse control difficulties | *7.394 (3,1461)* | *<.001* | 0.010 | T1-T0: –0.07 (–0.21 to 0.07)  *T2-T0: –0.28 (–0.42 to –0.14)*  *T3-T0: –0.27 (–0.42 to –0.12)* | 0.715 (1,1461) | .40 | .47 | <0.001 | –0.05 (–0.15 to 0.06) | 0.067 (3,1461) | .98 | >.99 | <0.001 |
| Difficulties engaging in goal-directed behavior | *23.565 (3,1461)* | *<.001* | 0.050 | *T1-T0: –0.43 (–0.57 to –0.29)*  *T2-T0: –0.51 (–0.66 to –0.37)*  *T3-T0: –0.49 (–0.65 to –0.34)* | 2.959 (1,1461) | .09 | .47 | 0.002 | –0.09 (–0.20 to 0.01) | 0.687 (3,1461) | .56 | >.99 | 0.001 |
| Limited access to emotion regulation strategies | *8.650 (3,1461)* | *<.001* | 0.020 | *T1-T0: –0.15 (–0.28 to –0.01)*  *T2-T0: –0.33 (–0.47 to –0.19)*  *T3-T0: –0.29 (–0.44 to –0.14)* | 2.077 (1,1461) | .15 | .47 | 0.001 | –0.08 (–0.18 to 0.03) | 0.763 (3,1461) | .51 | >.99 | 0.002 |
| **Social support** | *3.278 (3,1461)* | *.02* | 0.007 | T1-T0: 0.01 (–0.12 to 0.15)  T2-T0: 0.08 (–0.07 to 0.22)  *T3-T0: 0.22 (0.07 to 0.37)* | 2.960 (1,1461) | .09 | .17 | 0.002 | 0.09 (–0.01 to 0.20) | 1.719 (3,1461) | .16 | .31 | 0.004 |
| Subjective support | *3.706 (3,1461)* | *.01* | 0.008 | T1-T0: 0.08 (–0.06 to 0.21)  T2-T0: 0.12 (–0.02 to 0.26)  *T3-T0: 0.25 (0.10 to 0.40)* | 1.307 (1,1461) | .25 | .34 | 0.001 | 0.06 (–0.04 to 0.17) | 1.189 (3,1461) | .31 | .31 | 0.002 |
| Objective support | 2.198 (3,1461) | .09 | 0.004 | T1-T0: –0.14 (–0.28 to 0.00)  T2-T0: –0.05 (–0.19 to 0.09)  T3-T0: 0.04 (–0.11 to 0.19) | 0.500 (1,1461) | .48 | .48 | <0.001 | 0.04 (–0.07 to 0.14) | 1.266 (3,1461) | .28 | .31 | 0.003 |
| Support utilization | *5.356 (3,1461)* | *0.001* | 0.010 | T1-T0: 0.10 (–0.04 to 0.23)  T2-T0: 0.14 (–0.00 to 0.28)  *T3-T0: 0.30 (0.15 to 0.45)* | 4.731 (1,1461) | .03 | .12 | 0.003 | 0.12 (0.01 to 0.22) | 2.196 (3,1461) | .09 | .31 | 0.004 |

## **Table S3. Between-group comparisons for change scores in outcome indicators from baseline to postintervention, 1-month follow-up, and 3-month follow-up.** T0: baseline. T1: postintervention. T2: 1-month follow-up. T3: 3-month follow-up. *LSM*: least squares mean. *LSMD*: least squares mean difference. FDR: false discovery rate correction. At T1, T2, and T3, the intervention group included 189, 167, and 134 participants, while the control group included 208, 189, and 155 participants, respectively.

| **Measures** | **Timepoints** | **Intervention group, *LSM* (*SE*)** | **Control group, *LSM* (*SE*)** | ***LSMD* (95%CI)** | **Cohen *d* (95%CI)** | ***P* value** | **FDR adjusted *P* value** |
| --- | --- | --- | --- | --- | --- | --- | --- |
| **Depressive symptom severity** | T1-T0 | –4.98 (0.73) | –4.16 (0.70) | –0.82 (–2.13 to 0.50) | –0.13 (–0.33 to 0.08) | .22 | .66 |
|  | T2-T0 | –4.10 (0.72) | –4.31 (0.70) | 0.21 (–1.08 to 1.50) | 0.03 (–0.18 to 0.25) | .75 | .75 |
|  | T3-T0 | –3.83 (0.87) | –5.23 (0.83) | 1.40 (–0.08 to 2.88) | 0.22 (–0.01 to 0.45) | .06 | .19 |
| **Anxiety symptom severity** | T1-T0 | –2.23 (0.65) | –1.78 (0.63) | –0.45 (–1.62 to 0.74) | –0.08 (–0.28 to 0.13) | .46 | .69 |
|  | T2-T0 | –2.04 (0.70) | –2.71 (0.68) | 0.67 (–0.59 to 1.93) | 0.11 (–0.10 to 0.33) | .30 | .45 |
|  | T3-T0 | –2.20 (0.96) | –2.91 (0.94) | 0.71 (–0.69 to 2.12) | 0.12 (–0.12 to 0.36) | .32 | .47 |
| **Suicidal ideation severity** | T1-T0 | –2.82 (0.93) | –2.87 (0.90) | 0.05 (–1.63 to 1.73) | 0.01 (–0.19 to 0.21) | .95 | .95 |
|  | T2-T0 | –3.09 (0.88) | –3.94 (0.85) | 0.85 (–0.72 to 2.42) | 0.11 (–0.10 to 0.33) | .29 | .45 |
|  | T3-T0 | –3.37 (1.35) | –3.02 (1.32) | –0.35 (–2.33 to 1.62) | –0.04 (–0.28 to 0.19) | .72 | .72 |
| **Resilience** | T1-T0 | 6.11 (1.49) | 3.84 (1.44) | 2.27 (–0.43 to 4.96) | 0.17 (–0.03 to 0.37) | .10 | .20 |
|  | T2-T0 | 6.60 (1.82) | 4.01 (1.76) | 2.59 (–0.68 to 5.85) | 0.17 (–0.04 to 0.38) | .12 | .25 |
|  | T3-T0 | 5.32 (2.53) | 3.03 (2.47) | 2.29 (–1.40 to 5.98) | 0.15 (–0.09 to 0.38) | .22 | .76 |
| Emotion regulation | T1-T0 | 3.31 (0.58) | 2.06 (0.56) | 1.25 (0.20 to 2.30) | 0.24 (0.04 to 0.44) | .02 | .06 |
|  | T2-T0 | 2.67 (0.68) | 1.72 (0.66) | 0.95 (–0.26 to 2.17) | 0.17 (–0.05 to 0.38) | .12 | .25 |
|  | T3-T0 | 2.10 (0.93) | 1.49 (0.91) | 0.61 (–0.75 to 1.96) | 0.11 (–0.13 to 0.34) | .38 | .76 |
| Interpersonal support | T1-T0 | 2.03 (0.53) | 0.64 (0.52) | *1.39 (0.42 to 2.35)* | *0.29 (0.09 to 0.49)* | *.005* | *.03* |
|  | T2-T0 | 2.11 (0.59) | 0.97 (0.57) | 1.14 (0.08 to 2.19) | 0.23 (0.02 to 0.44) | .04 | .21 |
|  | T3-T0 | 0.13 (0.57) | 0.51 (0.55) | –0.38 (–1.39 to 0.63) | –0.08 (–0.29 to 0.13) | .46 | .76 |
| Family support | T1-T0 | 0.46 (0.47) | 0.73 (0.46) | –0.27 (–1.12 to 0.58) | –0.06 (–0.26 to 0.14) | .53 | .80 |
|  | T2-T0 | 0.66 (0.61) | 0.74 (0.59) | –0.08 (–1.17 to 1.01) | –0.02 (–0.23 to 0.20) | .89 | .89 |
|  | T3-T0 | –0.50 (1.09) | –0.56 (1.07) | 0.06 (–1.53 to 1.65) | 0.01 (–0.23 to 0.25) | .94 | .94 |
| Positive cognition | T1-T0 | –0.39 (0.47) | –0.43 (0.46) | 0.04 (–0.82 to 0.89) | 0.01 (–0.19 to 0.21) | .94 | .94 |
|  | T2-T0 | 0.12 (0.56) | –0.00 (0.54) | –0.12 (–1.12 to 0.87) | –0.03 (–0.24 to 0.19) | .81 | .89 |
|  | T3-T0 | 4.90 (0.83) | 5.19 (0.81) | –0.29 (–1.50 to 0.91) | –0.06 (–0.29 to 0.18) | .63 | .76 |
| Focused targeting | T1-T0 | 0.71 (0.61) | 0.84 (0.59) | –0.13 (–1.23 to 0.97) | –0.02 (–0.22 to 0.18) | .82 | .94 |
|  | T2-T0 | 1.04 (0.66) | 0.59 (0.63) | 0.45 (–0.72 to 1.62) | 0.08 (–0.13 to 0.29) | .45 | .68 |
|  | T3-T0 | 1.04 (0.90) | 0.67 (0.88) | 0.38 (–0.94 to 1.70) | 0.07 (–0.17 to 0.30) | .57 | .76 |
| **Difficulties in emotion regulation** | T1-T0 | –1.12 (2.29) | –0.51 (2.22) | –0.61 (–4.75 to 3.53) | –0.03 (–0.23 to 0.17) | .77 | .96 |
|  | T2-T0 | –4.99 (2.49) | –3.06 (2.41) | –1.93 (–6.38 to 2.53) | –0.09 (–0.30 to 0.12) | .40 | .84 |
|  | T3-T0 | –2.28 (3.69) | –1.34 (3.60) | –0.94 (–6.32 to 4.44) | –0.04 (–0.28 to 0.20) | .73 | .98 |
| Lack of emotional awareness | T1-T0 | –0.16 (0.74) | –0.10 (0.71) | –0.06 (–1.39 to 1.27) | –0.01 (–0.21 to 0.19) | .93 | .96 |
|  | T2-T0 | –0.98 (0.79) | –0.72 (0.77) | –0.26 (–1.68 to 1.16) | –0.04 (–0.25 to 0.17) | .72 | .89 |
|  | T3-T0 | –0.95 (1.07) | –1.15 (1.05) | 0.20 (–1.37 to 1.77) | 0.03 (–0.21 to 0.27) | .80 | .98 |
| Lack of emotional clarity | T1-T0 | 0.47 (0.41) | 0.58 (0.40) | –0.11 (–0.86 to 0.63) | –0.03 (–0.23 to 0.17) | .76 | .96 |
|  | T2-T0 | –0.23 (0.44) | –0.34 (0.42) | 0.10 (–0.68 to 0.89) | 0.03 (–0.18 to 0.24) | .80 | .89 |
|  | T3-T0 | 0.03 (0.65) | 0.02 (0.64) | 0.01 (–0.94 to 0.96) | 0.00 (–0.23 to 0.24) | .98 | .98 |
| Nonacceptance of emotional responses | T1-T0 | 0.51 (0.80) | 0.54 (0.78) | –0.03 (–1.49 to 1.42) | –0.00 (–0.21 to 0.20) | .96 | .96 |
|  | T2-T0 | –1.29 (0.89) | –0.71 (0.86) | –0.58 (–2.17 to 1.02) | –0.08 (–0.29 to 0.14) | .48 | .84 |
|  | T3-T0 | –0.46 (1.18) | –0.71 (1.15) | 0.25 (–1.47 to 1.98) | 0.03 (–0.20 to 0.27) | .77 | .98 |
| Impulse control difficulties | T1-T0 | 0.09 (0.62) | 0.12 (0.60) | –0.03 (–1.15 to 1.09) | –0.01 (–0.21 to 0.20) | .96 | .96 |
|  | T2-T0 | –1.00 (0.70) | –0.91 (0.68) | –0.09 (–1.35 to 1.16) | –0.02 (–0.23 to 0.20) | .89 | .89 |
|  | T3-T0 | –0.88 (0.99) | –0.99 (0.97) | 0.11 (–1.34 to 1.56) | 0.02 (–0.22 to 0.25) | .88 | .98 |
| Difficulties engaging in goal-directed behavior | T1-T0 | –1.65 (0.50) | –1.51 (0.49) | –0.14 (–1.05 to 0.76) | –0.03 (–0.23 to 0.17) | .75 | .96 |
|  | T2-T0 | –1.93 (0.58) | –1.38 (0.56) | –0.55 (–1.59 to 0.48) | –0.11 (–0.33 to 0.10) | .29 | .84 |
|  | T3-T0 | –1.00 (0.78) | –0.82 (0.77) | –0.18 (–1.32 to 0.97) | –0.04 (–0.27 to 0.20) | .76 | .98 |
| Limited access to emotion regulation strategies | T1-T0 | –0.38 (0.84) | –0.15 (0.81) | –0.23 (–1.75 to 1.29) | –0.03 (–0.23 to 0.17) | .77 | .96 |
|  | T2-T0 | –1.51 (0.90) | –0.45 (0.87) | –1.06 (–2.67 to 0.54) | –0.14 (–0.35 to 0.07) | .19 | .84 |
|  | T3-T0 | –0.92 (1.31) | 0.01 (1.28) | –0.93 (–2.85 to 0.99) | –0.11 (–0.35 to 0.12) | .34 | .98 |
| **Social support** | T1-T0 | 0.13 (0.10) | 0.05 (0.10) | 0.08 (–0.11 to 0.27) | 0.09 (–0.11 to 0.29) | .39 | .52 |
|  | T2-T0 | 0.13 (0.12) | –0.10 (0.12) | 0.23 (0.01 to 0.45) | 0.23 (0.01 to 0.44) | .04 | .07 |
|  | T3-T0 | 0.08 (0.17) | –0.06 (0.17) | 0.13 (–0.12 to 0.39) | 0.12 (–0.11 to 0.36) | .30 | .61 |
| Subjective support | T1-T0 | 0.18 (0.12) | 0.08 (0.11) | 0.10 (–0.11 to 0.32) | 0.10 (–0.11 to 0.30) | .35 | .52 |
|  | T2-T0 | 0.14 (0.14) | –0.08 (0.14) | 0.22 (–0.04 to 0.47) | 0.18 (–0.03 to 0.39) | .10 | .13 |
|  | T3-T0 | 0.05 (0.20) | –0.03 (0.20) | 0.08 (–0.22 to 0.38) | 0.06 (–0.17 to 0.30) | .59 | .69 |
| Objective support | T1-T0 | –0.04 (0.11) | –0.01 (0.11) | –0.03 (–0.23 to 0.18) | –0.03 (–0.23 to 0.17) | .78 | .78 |
|  | T2-T0 | –0.01 (0.13) | –0.17 (0.12) | 0.16 (–0.06 to 0.39) | 0.15 (–0.06 to 0.37) | .15 | .15 |
|  | T3-T0 | –0.03 (0.19) | –0.09 (0.19) | 0.06 (–0.22 to 0.33) | 0.05 (–0.19 to 0.28) | .69 | .69 |
| Support utilization | T1-T0 | 0.25 (0.12) | 0.07 (0.12) | 0.18 (–0.04 to 0.40) | 0.16 (–0.04 to 0.36) | .12 | .46 |
|  | T2-T0 | 0.27 (0.15) | –0.05 (0.14) | 0.32 (0.06 to 0.58) | 0.26 (0.04 to 0.47) | .02 | .07 |
|  | T3-T0 | 0.21 (0.20) | –0.04 (0.19) | 0.25 (–0.04 to 0.54) | 0.21 (–0.03 to 0.44) | .09 | .34 |

## **Table S4. Between-group differences in depression symptom remission rates across postintervention, 1-month follow-up, and 3-month follow-up.** T1: postintervention. T2: 1-month follow-up. T3: 3-month follow-up. OR: odds ratio.

| **Measures** | **Timepoints** | **Intervention group remission rate (%)** | **Control group remission rate (%)** | **Adjusted OR (95%CI)** | ***P* value** |
| --- | --- | --- | --- | --- | --- |
| **Full sample** | T1 | 105/189 (55.6%) | 102/208 (49.0%) | 1.21 (0.98 to 1.50) | .08 |
|  | T2 | 80/167 (47.9%) | 97/189 (51.3%) | 0.98 (0.78 to 1.22) | .84 |
|  | T3 | 58/134 (43.3%) | 88/155 (56.8%) | 0.79 (0.62 to 1.01) | .06 |
| **Subgroup with anxiety symptoms** | T1 | 53/123 (43.1%) | 39/128 (30.5%) | *1.39 (1.05 to 1.85)* | *.02* |
|  | T2 | 41/106 (38.7%) | 44/118 (37.3%) | 1.08 (0.98 to 1.45) | .61 |
|  | T3 | 27/83 (32.5%) | 38/94 (40.4%) | 0.82 (0.59 to 1.15) | .26 |
| **Subgroup without anxiety symptoms** | T1 | 52/66 (78.8%) | 63/80 (78.8%) | 1.05 (0.65 to 1.71) | .84 |
|  | T2 | 39/61 (63.9%) | 53/71 (74.6%) | 0.82 (0.52 to 1.27) | .37 |
|  | T3 | 31/51 (60.8%) | 50/61 (82.0%) | 0.64 (0.39 to 1.02) | .06 |

## **Table S5. Demographic characteristics and baseline levels of outcome indicators or the intervention and control groups in adolescents with and without anxiety symptoms.** ^a^ Family structure was classified into four types. Two-parent family: residing with both biological or adoptive parents; single-parent family: residing with only one parent; stepfamily: residing with one biological parent and a stepparent; no parent present: not living with either parent but staying with grandparents or other relatives.

| **Characters** | **Adolescents with anxiety symptoms** | | | | **Adolescents without anxiety symptoms** | | | |
| --- | --- | --- | --- | --- | --- | --- | --- | --- |
|  | **Intervention group (n=134)** | **Control group (n=141)** | ***t*/*χ*^2^ (df)** | ***P* value** | **Intervention group (n=78)** | **Control group (n=83)** | ***t*/*χ*^2^ (df)** | ***P* value** |
| **Age (years), mean (SD)** | 15.17 (1.41) | 14.89 (1.61) | 1.564 (271.21) | .12 | 14.88 (1.44) | 15.13 (1.46) | –1.085 (159) | .28 |
| **Gender, n (%)** |  |  | 0.006 (1) | .94 |  |  | 1.255 (1) | .26 |
| Male | 30 (22.4%) | 31 (22.0%) |  |  | 26 (33.3%) | 21 (25.3%) |  |  |
| Female | 104 (77.6%) | 110 (78.0%) |  |  | 52 (66.7%) | 62 (74.7%) |  |  |
| **Educational stage, n (%)** |  |  | 3.366 (1) | .07 |  |  | 2.628 (1) | .11 |
| Junior high school | 33 (24.6%) | 49 (34.8%) |  |  | 30 (38.5%) | 22 (26.5%) |  |  |
| Senior high school | 101 (75.4%) | 92 (65.2%) |  |  | 48 (61.5%) | 61 (73.5%) |  |  |
| **Only child status, n (%)** |  |  | 0.277 (1) | .60 |  |  | *4.707 (1)* | *.03* |
| Only child | 37 (27.6%) | 43 (30.5%) |  |  | 13 (16.7%) | 26 (31.3%) |  |  |
| Non-only child | 97 (72.4%) | 98 (70.5%) |  |  | 65 (83.3%) | 57 (68.7%) |  |  |
| **Family structure, n (%) ^a^** |  |  | 5.283 (3) | .15 |  |  | 1.427 (3) | .70 |
| Two‑parent family | 85 (63.4%) | 89 (63.1%) |  |  | 54 (69.2%) | 64 (77.1%) |  |  |
| Single‑parent family | 8 (6.0%) | 18 (12.8%) |  |  | 8 (10.3%) | 7 (8.4%) |  |  |
| Stepfamily | 15 (11.2%) | 9 (6.4%) |  |  | 2 (2.6%) | 2 (2.4%) |  |  |
| No parent present | 26 (19.4%) | 25 (17.7%) |  |  | 14 (17.9%) | 10 (12.0%) |  |  |
| **Socioeconomic status index, mean (SD)** | –0.14 (3.16) | 0.20 (2.97) | –0.911 (273) | .36 | –0.01 (2.77) | –0.12 (2.96) | 0.240 (159) | .81 |
| **Depressive symptom severity, mean (SD)** | 15.21 (3.04) | 14.94 (3.29) | 0.712 (273) | .48 | 13.60 (2.67) | 13.11 (2.11) | 1.308 (159) | .19 |
| **Anxiety symptom severity, mean (SD)** | 14.36 (3.31) | 14.58 (3.16) | –0.572 (273) | .57 | 6.41 (2.46) | 5.87 (2.92) | 1.271 (159) | .21 |
| **Suicidal ideation severity, mean (SD)** | 15.63 (6.91) | 15.60 (7.80) | 0.043 (273) | .97 | 9.77 (7.41) | 8.54 (8.27) | 0.989 (159) | .32 |
| **Resilience, mean (SD)** | 68.06 (14.42) | 68.14 (14.82) | –0.043 (273) | .97 | 79.58 (13.53) | 83.28 (17.79) | –1.491 (152.47) | .14 |
| Emotion regulation, mean (SD) | 12.38 (3.93) | 12.21 (4.38) | 0.330 (273) | .74 | 16.64 (4.33) | 17.64 (5.29) | –1.314 (156.11) | .19 |
| Interpersonal support, mean (SD) | 13.55 (4.58) | 14.36 (4.68) | –1.438 (273) | .15 | 17.78 (4.47) | 18.65 (5.39) | –1.109 (159) | .27 |
| Family support, mean (SD) | 16.10 (5.29) | 15.56 (5.25) | 0.860 (273) | .39 | 17.44 (4.45) | 18.16 (5.07) | –0.956 (159) | .34 |
| Positive cognition, mean (SD) | 12.59 (3.45) | 12.37 (3.63) | 0.509 (273) | .61 | 12.86 (3.34) | 13.41 (3.61) | –1.003 (159) | .32 |
| Focused targeting, mean (SD) | 13.43 (4.41) | 13.64 (4.20) | –0.390 (273) | .70 | 14.86 (4.18) | 15.42 (4.56) | –0.815 (159) | .42 |
| **Difficulties in emotion regulation, mean (SD)** | 120.79 (16.55) | 121.60 (18.04) | –0.388 (273) | .70 | 101.78 (14.95) | 102.02 (19.66) | –0.088 (152.48) | .93 |
| Lack of emotional awareness, mean (SD) | 16.18 (4.63) | 15.81 (4.47) | 0.676 (273) | .50 | 16.60 (5.04) | 16.33 (5.04) | 0.35 (159) | .73 |
| Lack of emotional clarity, mean (SD) | 16.18 (3.39) | 16.47 (3.55) | –0.690 (273) | .49 | 13.99 (2.61) | 13.66 (3.60) | 0.657 (149.53) | .51 |
| Nonacceptance of emotional responses, mean (SD) | 20.51 (4.82) | 20.77 (4.75) | –0.448 (273) | .66 | 16.36 (5.13) | 16.71 (5.79) | –0.407 (159) | .68 |
| Impulse control difficulties, mean (SD) | 20.61 (4.64) | 21.09 (5.14) | –0.812 (273) | .42 | 16.49 (4.39) | 17.11 (4.83) | –0.85 (159) | .40 |
| Difficulties engaging in goal-directed behavior, mean (SD) | 18.63 (3.91) | 18.85 (4.00) | –0.454 (273) | .65 | 15.67 (3.94) | 15.93 (4.71) | –0.380 (159) | .70 |
| Limited access to emotion regulation strategies, mean (SD) | 28.68 (5.52) | 28.62 (6.20) | 0.088 (273) | .93 | 22.68 (5.96) | 22.29 (6.68) | 0.390 (159) | .70 |
| **Social support, mean (SD)** | 2.87 (0.97) | 2.87 (1.00) | –0.074 (273) | .94 | 3.35 (0.76) | 3.67 (0.85) | *–2.535 (159)* | *.01* |
| Subjective support, mean (SD) | 2.83 (1.21) | 2.84 (1.14) | –0.131 (273) | .90 | 3.33 (0.86) | 3.60 (0.94) | –1.853 (159) | .07 |
| Objective support, mean (SD) | 3.12 (1.08) | 3.06 (1.08) | 0.406 (273) | .69 | 3.49 (0.88) | 3.80 (0.88) | *–2.276 (159)* | *.02* |
| Support utilization, mean (SD) | 2.65 (1.09) | 2.71 (1.14) | –0.463 (273) | .64 | 3.22 (0.91) | 3.59 (1.09) | *–2.365 (159)* | *.02* |

## **Table S6. Estimated means and standard errors for continuous outcome indicators at each time point for the intervention and control groups stratified by adolescent anxiety symptom status (adjusted for baseline covariates using analysis of covariance).** For adolescents with anxiety symptoms, the intervention group included 134, 123, 106, and 83 participants at baseline, postintervention, 1-month follow-up, and 3-month follow-up, while the control group included 141, 128, 118, and 95 participants. For adolescents without anxiety symptoms, the intervention group included 78, 66, 61, and 51 participants at different time points, while the control group included 83, 80, 71, and 61 participants. ^a^ Due to baseline imbalances in objective support, support utilization, and overall social support between the intervention and control groups, estimated means and standard errors of these indicators were not calculated.

| **Outcome indicator** | **Group** | **Adolescents with anxiety symptoms** | | | | **Adolescents without anxiety symptoms** | | | |
| --- | --- | --- | --- | --- | --- | --- | --- | --- | --- |
|  |  | **Baseline** | **Postintervention** | **1-month follow-up** | **3-month follow-up** | **Baseline** | **Postintervention** | **1-month follow-up** | **3-month follow-up** |
| **Depressive symptom severity, mean (SE)** | Intervention | 15.20 (0.55) | 11.20 (0.57) | 11.60 (0.61) | 12.00 (0.68) | 13.54 (0.61) | 5.98 (0.65) | 6.72 (0.67) | 7.90 (0.73) |
|  | Control | 15.00 (0.53) | 12.50 (0.55) | 11.40 (0.57) | 10.80 (0.64) | 13.71 (0.62) | 6.23 (0.62) | 6.82 (0.65) | 6.46 (0.69) |
| **Anxiety symptom severity, mean (SE)** | Intervention | 14.80 (0.52) | 10.80 (0.54) | 10.90 (0.57) | 10.40 (0.64) | 6.91 (0.54) | 6.11 (0.58) | 5.95 (0.59) | 6.21 (0.65) |
|  | Control | 14.80 (0.50) | 11.30 (0.52) | 10.20 (0.54) | 9.60 (0.60) | 6.85 (0.54) | 5.91 (0.55) | 5.76 (0.57) | 5.87 (0.61) |
| **Suicidal ideation severity, mean (SE)** | Intervention | 16.00 (0.69) | 13.30 (0.72) | 12.60 (0.76) | 10.60 (0.85) | 9.73 (0.80) | 7.00 (0.86) | 5.97 (0.89) | 6.46 (0.96) |
|  | Control | 15.80 (0.66) | 12.70 (0.69) | 11.40 (0.72) | 10.60 (0.80) | 9.20 (0.81) | 6.85 (0.82) | 5.87 (0.86) | 6.74 (0.90) |
| **Resilience, mean (SE)** | Intervention | 67.60 (1.11) | 77.30 (1.16) | 76.80 (1.23) | 77.00 (1.37) | 81.30 (1.50) | 82.10 (1.61) | 85.20 (1.66) | 83.30 (1.80) |
|  | Control | 67.60 (1.07) | 74.80 (1.11) | 75.40 (1.15) | 75.80 (1.29) | 81.60 (1.52) | 81.50 (1.54) | 81.40 (1.60) | 80.80 (1.69) |
| Emotion regulation | Intervention | 12.00 (0.45) | 15.70 (0.47) | 15.50 (0.49) | 16.60 (0.55) | 16.90 (0.55) | 19.60 (0.59) | 19.50 (0.61) | 18.40 (0.66) |
|  | Control | 12.00 (0.43) | 14.50 (0.45) | 14.90 (0.46) | 16.40 (0.52) | 17.20 (0.56) | 18.80 (0.57) | 18.30 (0.59) | 17.80 (0.62) |
| Interpersonal support | Intervention | 13.70 (0.38) | 16.80 (0.40) | 17.00 (0.42) | 12.60 (0.47) | 18.10 (0.51) | 18.90 (0.55) | 19.00 (0.57) | 13.00 (0.62) |
|  | Control | 14.10 (0.37) | 15.40 (0.38) | 16.40 (0.40) | 12.00 (0.44) | 18.30 (0.52) | 18.50 (0.52) | 18.00 (0.55) | 11.60 (0.58) |
| Family support | Intervention | 15.80 (0.41) | 17.00 (0.43) | 17.10 (0.45) | 16.10 (0.50) | 17.60 (0.52) | 17.60 (0.56) | 18.00 (0.58) | 18.70 (0.63) |
|  | Control | 15.60 (0.39) | 17.00 (0.41) | 16.90 (0.42) | 15.90 (0.47) | 17.50 (0.53) | 17.70 (0.54) | 17.80 (0.56) | 18.30 (0.59) |
| Positive cognition | Intervention | 12.60 (0.36) | 13.10 (0.37) | 12.70 (0.39) | 17.70 (0.44) | 13.30 (0.49) | 11.60 (0.52) | 12.70 (0.54) | 17.90 (0.58) |
|  | Control | 12.50 (0.34) | 12.80 (0.36) | 12.80 (0.37) | 17.20 (0.41) | 13.20 (0.49) | 12.00 (0.50) | 12.30 (0.52) | 18.90 (0.55) |
| Focused targeting | Intervention | 13.50 (0.42) | 14.90 (0.44) | 14.50 (0.46) | 14.10 (0.52) | 15.70 (0.61) | 14.60 (0.65) | 16.20 (0.67) | 15.50 (0.73) |
|  | Control | 13.60 (0.40) | 15.20 (0.42) | 14.50 (0.43) | 14.40 (0.48) | 15.60 (0.61) | 14.70 (0.62) | 15.20 (0.65) | 14.40 (0.68) |
| **Difficulties in emotion regulation, mean (SE)** | Intervention | 122.00 (1.71) | 116.00 (1.79) | 113.00 (1.89) | 113.00 (2.11) | 101.60 (2.26) | 105.10 (2.43) | 98.80 (2.51) | 100.70 (2.72) |
|  | Control | 122.00 (1.64) | 119.00 (1.71) | 116.00 (1.78) | 115.00 (1.98) | 102.50 (2.28) | 103.40 (2.31) | 100.20 (2.41) | 101.90 (2.54) |
| Lack of emotional awareness | Intervention | 15.90 (0.50) | 15.60 (0.52) | 17.10 (0.55) | 16.70 (0.62) | 16.00 (0.72) | 18.10 (0.78) | 17.50 (0.80) | 17.40 (0.87) |
|  | Control | 15.70 (0.48) | 15.90 (0.50) | 16.40 (0.52) | 16.80 (0.58) | 15.90 (0.73) | 17.50 (0.74) | 17.30 (0.77) | 16.60 (0.82) |
| Lack of emotional clarity | Intervention | 16.20 (0.28) | 15.90 (0.29) | 15.40 (0.31) | 15.60 (0.35) | 13.80 (0.37) | 14.80 (0.39) | 13.60 (0.41) | 14.30 (0.44) |
|  | Control | 16.30 (0.27) | 16.10 (0.28) | 15.50 (0.29) | 15.50 (0.33) | 13.80 (0.37) | 14.70 (0.38) | 13.90 (0.39) | 14.70 (0.41) |
| Nonacceptance of emotional responses | Intervention | 20.60 (0.57) | 20.20 (0.60) | 18.50 (0.63) | 18.80 (0.70) | 16.60 (0.76) | 17.00 (0.82) | 15.90 (0.84) | 16.00 (0.92) |
|  | Control | 20.80 (0.55) | 20.70 (0.57) | 19.80 (0.59) | 18.90 (0.66) | 17.10 (0.77) | 16.70 (0.78) | 16.20 (0.81) | 16.50 (0.86) |
| Impulse control difficulties | Intervention | 20.90 (0.45) | 20.20 (0.47) | 18.90 (0.50) | 19.40 (0.56) | 16.70 (0.60) | 17.20 (0.64) | 16.60 (0.67) | 16.40 (0.72) |
|  | Control | 21.10 (0.44) | 20.60 (0.45) | 19.90 (0.47) | 19.40 (0.52) | 17.20 (0.60) | 17.10 (0.61) | 16.10 (0.64) | 16.60 (0.67) |
| Difficulties engaging in goal-directed behavior | Intervention | 18.90 (0.36) | 16.90 (0.38) | 16.50 (0.40) | 16.80 (0.45) | 15.80 (0.49) | 14.80 (0.53) | 13.90 (0.55) | 14.40 (0.59) |
|  | Control | 18.90 (0.35) | 17.40 (0.37) | 17.40 (0.38) | 17.00 (0.42) | 16.00 (0.50) | 14.70 (0.50) | 14.60 (0.53) | 14.70 (0.55) |
| Limited access to emotion regulation strategies | Intervention | 29.20 (0.61) | 27.40 (0.64) | 26.00 (0.67) | 26.10 (0.75) | 22.50 (0.82) | 23.00 (0.88) | 21.00 (0.91) | 21.90 (0.98) |
|  | Control | 29.10 (0.59) | 27.90 (0.61) | 27.20 (0.63) | 27.00 (0.71) | 22.60 (0.82) | 22.60 (0.84) | 22.10 (0.87) | 22.60 (0.92) |
| **Social support, mean (SE)** | Intervention | 2.89 (0.08) | 2.90 (0.08) | 3.02 (0.09) | 3.25 (0.10) | - ^a^ | - | - | - |
|  | Control | 2.91 (0.08) | 2.86 (0.08) | 2.99 (0.08) | 3.11 (0.09) | - | - | - | - |
| Subjective support | Intervention | 2.85 (0.09) | 2.93 (0.10) | 3.02 (0.10) | 3.23 (0.11) | 3.40 (0.12) | 3.59 (0.13) | 3.72 (0.13) | 3.50 (0.14) |
|  | Control | 2.87 (0.09) | 2.85 (0.09) | 2.93 (0.10) | 3.12 (0.11) | 3.48 (0.12) | 3.54 (0.12) | 3.31 (0.13) | 3.52 (0.13) |
| Objective support | Intervention | 3.16 (0.09) | 2.96 (0.09) | 3.15 (0.09) | 3.33 (0.11) | - | - | - | - |
|  | Control | 3.16 (0.08) | 3.04 (0.09) | 3.20 (0.09) | 3.30 (0.10) | - | - | - | - |
| Support utilization | Intervention | 2.67 (0.09) | 2.82 (0.09) | 2.90 (0.10) | 3.20 (0.11) | - | - | - | - |
|  | Control | 2.70 (0.09) | 2.71 (0.09) | 2.86 (0.09) | 2.95 (0.10) | - | - | - | - |

## **Table S7. Between-group comparisons for scores of outcome indicators at postintervention, 1-month follow-up, and 3-month follow-up assessments in adolescents with anxiety symptoms.** T0: baseline. T1: postintervention. T2: 1-month follow-up. T3: 3-month follow-up. FDR: false discovery rate correction.

| **Measures** | **Time effect** | | | | **Group effect** | | | | | **Time-by-group interaction** | | | |
| --- | --- | --- | --- | --- | --- | --- | --- | --- | --- | --- | --- | --- | --- |
|  | ***F* (df)** | ***P* value** | **η^2^_p_** | **Post hoc comparisons Cohen *d* (95%CI)** | ***F* (df)** | ***P* value** | **FDR adjusted *P* value** | **η^2^_p_** | **Between-group comparisons (intervention group-control group) Cohen *d* (95%CI)** | ***F* (df)** | ***P* value** | **FDR adjusted *P* value** | **η^2^_p_** |
| **Depressive symptom severity** | *26.971 (3,910)* | *<.001* | 0.080 | *T1-T0: –0.60 (–0.78 to –0.43)*  *T2-T0: –0.66 (–0.84 to –0.48)*  *T3-T0: –0.69 (–0.88 to –0.49)* | 0.009 (1,910) | .92 | - | <0.001 | 0.01 (–0.13 to 0.14) | 2.073 (3,910) | .10 | - | 0.007 |
| **Anxiety symptom severity** | *45.983 (3,910)* | *<.001* | 0.130 | *T1-T0: –0.74 (–0.92 to –0.57)*  *T2-T0: –0.84 (–1.02 to –0.66)*  *T3-T0: –0.96 (–1.15 to –0.76)* | 0.374 (1,910) | .54 | .54 | <0.001 | 0.04 (–0.09 to 0.18) | 0.854 (3,910) | .46 | .78 | 0.003 |
| **Suicidal ideation severity** | *24.961 (3,910)* | *<.001* | 0.080 | *T1-T0: –0.43 (–0.60 to –0.26)*  *T2-T0: –0.57 (–0.75 to –0.39)*  *T3-T0: –0.78 (–0.97 to –0.58)* | 1.189 (1,910) | .28 | .54 | 0.001 | 0.07 (–0.06 to 0.21) | 0.369 (3,910) | .78 | .78 | 0.001 |
| **Resilience** | *39.824 (3,909)* | *<.001* | 0.120 | *T1-T0: 0.78 (0.61 to 0.96)*  *T2-T0: 0.78 (0.60 to 0.96)*  *T3-T0: 0.81 (0.61 to 1.00)* | 2.916 (1,909) | .09 | .18 | 0.003 | 0.12 (–0.02 to 0.25) | 0.576 (3,909) | .63 | .97 | 0.002 |
| Emotion regulation | *45.440 (3,909)* | *<.001* | 0.130 | *T1-T0: 0.71 (0.54 to 0.89)*  *T2-T0: 0.73 (0.55 to 0.91)*  *T3-T0: 1.03 (0.84 to 1.23)* | 3.011 (1,909) | .08 | .18 | 0.003 | 0.12 (–0.02 to 0.25) | 0.860 (3,909) | .46 | .97 | 0.003 |
| Interpersonal support | *60.682 (3,909)* | *<.001* | 0.170 | *T1-T0: 0.59 (0.41 to 0.76)*  *T2-T0: 0.75 (0.57 to 0.93)*  T3-T0: 0.16 (–0.02 to 0.34) | 4.715 (1,909) | .03 | .18 | 0.005 | 0.15 (0.01 to 0.28) | 2.616 (3,909) | .05 | .30 | 0.009 |
| Family support | *7.012 (3,909)* | *<.001* | 0.020 | *T1-T0: 0.32 (0.15 to 0.50)*  *T2-T0: 0.32 (0.15 to 0.50)*  T3-T0: 0.06 (–0.13 to 0.25) | 0.240 (1,909) | .62 | .62 | <0.001 | 0.03 (–0.10 to 0.17) | 0.076 (3,909) | .97 | .97 | <0.001 |
| Positive cognition | *84.802 (3,909)* | *<.001* | 0.220 | T1-T0: 0.11 (–0.06 to 0.28)  T2-T0: 0.06 (–0.12 to 0.23)  *T3-T0: 1.39 (1.19 to 1.59)* | 0.512 (1,909) | .47 | .62 | 0.001 | 0.05 (–0.09 to 0.18) | 0.227 (3,909) | .88 | .97 | 0.001 |
| Focused targeting | *6.204 (3,909)* | *<.001* | 0.020 | *T1-T0: 0.37 (0.20 to 0.54)*  *T2-T0: 0.24 (0.06 to 0.42)*  T3-T0: 0.18 (–0.01 to 0.37) | 0.373 (1,909) | .54 | .62 | <0.001 | –0.04 (–0.18 to 0.09) | 0.131 (3,909) | .94 | .97 | <0.001 |
| **Difficulties in emotion regulation** | *11.087 (3,910)* | *<.001* | 0.040 | *T1-T0: –0.26 (–0.43 to –0.09)*  *T2-T0: –0.45 (–0.62 to –0.27)*  *T3-T0: –0.46 (–0.65 to –0.27)* | 2.607 (1,910) | .11 | .27 | 0.003 | –0.11 (–0.24 to 0.02) | 0.499 (3,910) | .68 | .85 | 0.002 |
| Lack of emotional awareness | *2.913 (3,910)* | *.03* | 0.010 | T1-T0: –0.01 (–0.18 to 0.16)  *T2-T0: –0.19 (–0.37 to –0.02)*  *T3-T0: –0.19 (–0.38 to –0.00)* | 0.069 (1,910) | .79 | .79 | <0.001 | –0.02 (–0.15 to 0.12) | 0.491 (3,910) | .69 | .85 | 0.002 |
| Lack of emotional clarity | *4.569 (3,910)* | *.003* | 0.010 | T1-T0: –0.08 (–0.25 to 0.09)  *T2-T0: –0.29 (–0.47 to –0.11)*  *T3-T0: –0.26 (–0.45 to –0.07)* | 0.241 (1,910) | .62 | .73 | <0.001 | –0.03 (–0.17 to 0.10) | 0.137 (3,910) | .94 | .94 | <0.001 |
| Nonacceptance of emotional responses | *6.068 (3,910)* | *<.001* | 0.020 | T1-T0: –0.04 (–0.21 to 0.13)  *T2-T0: –0.28 (–0.45 to –0.10)*  *T3-T0: –0.33 (–0.52 to –0.14)* | 2.033 (1,910) | .15 | .27 | 0.002 | –0.10 (–0.23 to 0.04) | 0.608 (3,910) | .61 | .85 | 0.002 |
| Impulse control difficulties | *7.393 (3,910)* | *<.001* | 0.020 | T1-T0: –0.13 (–0.31 to 0.04)  *T2-T0: –0.36 (–0.54 to –0.18)*  *T3-T0: –0.36 (–0.55 to –0.17)* | 1.712 (1,910) | .19 | .27 | 0.002 | –0.09 (–0.22 to 0.05) | 0.441 (3,910) | .72 | .85 | 0.001 |
| Difficulties engaging in goal-directed behavior | *17.924 (3,910)* | *<.001* | 0.060 | *T1-T0: –0.49 (–0.66 to –0.31)*  *T2-T0: –0.54 (–0.72 to –0.36)*  *T3-T0: –0.56 (–0.75 to –0.37)* | 2.415 (1,910) | .12 | .27 | 0.003 | –0.11 (–0.24 to 0.03) | 0.648 (3,910) | .58 | .85 | 0.002 |
| Limited access to emotion regulation strategies | *9.979 (3,910)* | *<.001* | 0.030 | *T1-T0: –0.25 (–0.43 to –0.08)*  *T2-T0: –0.43 (–0.61 to –0.25)*  *T3-T0: –0.43 (–0.62 to –0.24)* | 2.327 (1,910) | .13 | .27 | 0.003 | –0.10 (–0.24 to 0.03) | 0.549 (3,910) | .65 | .85 | 0.002 |
| **Social support** | *6.400 (3,910)* | *<.001* | 0.020 | T1-T0: –0.03 (–0.20 to 0.15)  T2-T0: 0.14 (–0.04 to 0.32)  *T3-T0: 0.37 (0.17 to 0.56)* | 0.685 (1,910) | .41 | .54 | 0.001 | 0.06 (–0.08 to 0.19) | 0.344 (3,910) | .79 | .90 | 0.001 |
| Subjective support | *4.888 (3,910)* | *.002* | 0.020 | T1-T0: 0.04 (–0.14 to 0.21)  T2-T0: 0.13 (–0.04 to 0.31)  *T3-T0: 0.35 (0.16 to 0.54)* | 1.183 (1,910) | .28 | .54 | 0.001 | 0.07 (–0.06 to 0.21) | 0.226 (3,910) | .88 | .90 | 0.001 |
| Objective support | *4.994 (3,910)* | *.002* | 0.020 | *T1-T0: 0.19 (0.02 to 0.36)*  T2-T0: 0.02 (–0.16 to 0.19)  T3-T0: 0.18 (–0.01 to 0.37) | 0.134 (1,910) | .71 | .72 | <0.001 | –0.03 (–0.16 to 0.11) | 0.190 (3,910) | .90 | .90 | 0.001 |
| Support utilization | *7.753 (3,910)* | *<.001* | 0.020 | T1-T0: 0.09 (–0.08 to 0.26)  *T2-T0: 0.22 (0.04 to 0.40)*  *T3-T0: 0.45 (0.26 to 0.64)* | 2.371 (1,910) | .12 | .50 | 0.003 | 0.11 (–0.03 to 0.24) | 0.996 (3,910) | .39 | .90 | 0.003 |

## **Table S8. Between-group comparisons for change scores in outcome indicators from baseline to postintervention, 1-month follow-up, and 3-month follow-up among adolescents with anxiety symptoms.** T0: baseline. T1: postintervention. T2: 1-month follow-up. T3: 3-month follow-up. *LSM*: least squares mean. *LSMD*: least squares mean difference. FDR: false discovery rate correction. At T1, T2, and T3, the intervention group included 123, 106, and 83 participants while the control group included 128, 118, and 95 participants, respectively.

| **Measures** | **Timepoints** | **Intervention group, *LSM* (*SE*)** | **Control group, *LSM* (*SE*)** | ***LSMD* (95%CI)** | **Cohen *d* (95%CI)** | ***P* value** | **FDR adjusted *P* value** |
| --- | --- | --- | --- | --- | --- | --- | --- |
| **Depressive symptom severity** | T1-T0 | –3.83 (0.94) | –2.36 (0.87) | –1.47 (–3.19 to 0.26) | –0.22 (–0.48 to 0.04) | .10 | .29 |
|  | T2-T0 | –3.55 (0.90) | –3.67 (0.83) | 0.12 (–1.52 to 1.77) | 0.02 (–0.25 to 0.29) | .88 | .88 |
|  | T3-T0 | –3.27 (1.41) | –4.52 (1.34) | 1.25 (–0.80 to 3.31) | 0.19 (–0.12 to 0.50) | .23 | .49 |
| **Anxiety symptom severity** | T1-T0 | –3.24 (0.89) | –2.66 (0.83) | –0.58 (–2.21 to 1.05) | –0.09 (–0.35 to 0.17) | .48 | .69 |
|  | T2-T0 | –3.66 (0.91) | –4.44 (0.85) | 0.78 (–0.90 to 2.45) | 0.13 (–0.15 to 0.40) | .36 | .54 |
|  | T3-T0 | –4.45 (1.26) | –5.37 (1.20) | 0.92 (–0.92 to 2.77) | 0.15 (–0.16 to 0.46) | .33 | .49 |
| **Suicidal ideation severity** | T1-T0 | –2.08 (1.20) | –2.52 (1.12) | 0.44 (–1.77 to 2.65) | 0.05 (–0.21 to 0.31) | .69 | .69 |
|  | T2-T0 | –2.52 (1.12) | –4.09 (1.04) | 1.57 (–0.49 to 3.63) | 0.21 (–0.06 to 0.48) | .13 | .40 |
|  | T3-T0 | –3.57 (1.87) | –3.59 (1.78) | 0.02 (–2.70 to 2.75) | 0.00 (–0.31 to 0.31) | .99 | .99 |
| **Resilience** | T1-T0 | 9.27 (1.87) | 6.71 (1.74) | 2.56 (–0.87 to 5.99) | 0.19 (–0.07 to 0.45) | .14 | .29 |
|  | T2-T0 | 7.90 (2.07) | 6.73 (1.92) | 1.17 (–2.62 to 4.98) | 0.08 (–0.19 to 0.36) | .54 | .90 |
|  | T3-T0 | 7.94 (3.21) | 6.70 (3.06) | 1.24 (–3.45 to 5.93) | 0.08 (–0.23 to 0.39) | .60 | .81 |
| Emotion regulation | T1-T0 | 3.69 (0.72) | 2.43 (0.67) | 1.26 (–0.07 to 2.58) | 0.24 (–0.02 to 0.50) | .06 | .19 |
|  | T2-T0 | 3.17 (0.83) | 2.60 (0.77) | 0.57 (–0.97 to 2.10) | 0.10 (–0.17 to 0.37) | .47 | .90 |
|  | T3-T0 | 2.73 (1.25) | 2.42 (1.19) | 0.31 (–1.52 to 2.13) | 0.05 (–0.26 to 0.36) | .74 | .81 |
| Interpersonal support | T1-T0 | 2.75 (0.69) | 0.96 (0.64) | *1.79 (0.53 to 3.06)* | *0.37 (0.11 to 0.63)* | *.006* | *.04* |
|  | T2-T0 | 3.09 (0.71) | 1.95 (0.66) | 1.14 (–0.17 to 2.45) | 0.24 (–0.04 to 0.51) | .09 | .53 |
|  | T3-T0 | 0.16 (0.73) | 1.08 (0.67) | –0.92 (–2.26 to 0.41) | –0.19 (–0.46 to 0.08) | .17 | .81 |
| Family support | T1-T0 | 1.03 (0.59) | 1.40 (0.55) | –0.37 (–1.45 to 0.72) | –0.09 (–0.35 to 0.17) | .50 | .76 |
|  | T2-T0 | 0.79 (0.73) | 1.14 (0.68) | –0.35 (–1.70 to 0.99) | –0.07 (–0.35 to 0.20) | .60 | .90 |
|  | T3-T0 | –1.02 (1.50) | –0.75 (1.43) | –0.26 (–2.45 to 1.92) | –0.04 (–0.35 to 0.27) | .81 | .81 |
| Positive cognition | T1-T0 | 0.22 (0.59) | 0.08 (0.55) | 0.14 (–0.94 to 1.22) | 0.03 (–0.22 to 0.29) | .80 | .80 |
|  | T2-T0 | 0.17 (0.68) | 0.32 (0.63) | –0.16 (–1.40 to 1.09) | –0.03 (–0.31 to 0.24) | .81 | .97 |
|  | T3-T0 | 5.60 (1.07) | 5.37 (1.02) | 0.23 (–1.34 to 1.79) | 0.04 (–0.26 to 0.35) | .78 | .81 |
| Focused targeting | T1-T0 | 1.57 (0.75) | 1.83 (0.70) | –0.26 (–1.64 to 1.12) | –0.05 (–0.31 to 0.21) | .71 | .80 |
|  | T2-T0 | 0.69 (0.79) | 0.71 (0.74) | –0.02 (–1.47 to 1.44) | –0.00 (–0.28 to 0.27) | .98 | .98 |
|  | T3-T0 | 1.14 (1.09) | 1.43 (1.04) | –0.29 (–1.88 to 1.31) | –0.06 (–0.36 to 0.25) | .72 | .81 |
| **Difficulties in emotion regulation** | T1-T0 | –2.51 (2.89) | –0.74 (2.69) | –1.77 (–7.07 to 3.53) | –0.09 (–0.34 to 0.17) | .51 | .84 |
|  | T2-T0 | –8.63 (3.08) | –5.44 (2.86) | –3.19 (–8.85 to 2.47) | –0.15 (–0.43 to 0.12) | .27 | .53 |
|  | T3-T0 | –7.70 (4.69) | –7.10 (4.47) | –0.60 (–7.45 to 6.25) | –0.03 (–0.34 to 0.28) | .86 | .91 |
| Lack of emotional awareness | T1-T0 | –0.80 (0.91) | –0.53 (0.84) | –0.27 (–1.93 to 1.40) | –0.04 (–0.30 to 0.22) | .75 | .84 |
|  | T2-T0 | –1.47 (0.98) | –0.99 (0.91) | –0.48 (–2.28 to 1.31) | –0.07 (–0.35 to 0.20) | .60 | .60 |
|  | T3-T0 | –0.61 (1.35) | –1.22 (1.29) | 0.61 (–1.37 to 2.58) | 0.09 (–0.21 to 0.40) | .55 | .91 |
| Lack of emotional clarity | T1-T0 | 0.16 (0.54) | 0.27 (0.50) | –0.10 (–1.10 to 0.89) | –0.03 (–0.28 to 0.23) | .84 | .84 |
|  | T2-T0 | –0.34 (0.56) | –0.63 (0.52) | 0.29 (–0.73 to 1.31) | 0.08 (–0.20 to 0.35) | .58 | .60 |
|  | T3-T0 | –1.39 (0.86) | –1.73 (0.82) | 0.34 (–0.93 to 1.60) | 0.08 (–0.23 to 0.39) | .60 | .91 |
| Nonacceptance of emotional responses | T1-T0 | 0.37 (1.03) | 0.92 (0.96) | –0.55 (–2.44 to 1.33) | –0.08 (–0.33 to 0.18) | .57 | .84 |
|  | T2-T0 | –2.88 (1.10) | –1.46 (1.02) | –1.41 (–3.43 to 0.60) | –0.19 (–0.46 to 0.08) | .17 | .53 |
|  | T3-T0 | –2.03 (1.54) | –2.31 (1.46) | 0.29 (–1.96 to 2.53) | 0.04 (–0.27 to 0.35) | .80 | .91 |
| Impulse control difficulties | T1-T0 | –0.13 (0.76) | 0.05 (0.71) | –0.18 (–1.57 to 1.22) | –0.03 (–0.29 to 0.22) | .80 | .84 |
|  | T2-T0 | –2.02 (0.88) | –1.30 (0.82) | –0.72 (–2.34 to 0.89) | –0.12 (–0.39 to 0.15) | .38 | .53 |
|  | T3-T0 | –2.01 (1.30) | –2.30 (1.24) | 0.30 (–1.61 to 2.20) | 0.05 (–0.26 to 0.36) | .76 | .91 |
| Difficulties engaging in goal-directed behavior | T1-T0 | –1.48 (0.62) | –1.30 (0.58) | –0.18 (–1.32 to 0.96) | –0.04 (–0.30 to 0.22) | .76 | .84 |
|  | T2-T0 | –2.42 (0.70) | –1.80 (0.65) | –0.62 (–1.91 to 0.66) | –0.13 (–0.40 to 0.14) | .34 | .53 |
|  | T3-T0 | –1.04 (0.99) | –0.95 (0.94) | –0.09 (–1.53 to 1.35) | –0.02 (–0.33 to 0.29) | .91 | .91 |
| Limited access to emotion regulation strategies | T1-T0 | –0.63 (1.07) | –0.14 (0.99) | –0.49 (–2.45 to 1.47) | –0.06 (–0.32 to 0.19) | .62 | .84 |
|  | T2-T0 | –2.44 (1.10) | –1.24 (1.02) | –1.20 (–3.23 to 0.82) | –0.16 (–0.43 to 0.11) | .24 | .53 |
|  | T3-T0 | –1.86 (1.61) | –1.03 (1.53) | –0.83 (–3.18 to 1.52) | –0.11 (–0.42 to 0.20) | .49 | .91 |
| **Social support** | T1-T0 | 0.02 (0.14) | 0.01 (0.13) | 0.01 (–0.25 to 0.27) | 0.01 (–0.25 to 0.27) | .95 | .95 |
|  | T2-T0 | 0.11 (0.14) | 0.10 (0.13) | 0.01 (–0.25 to 0.26) | 0.01 (–0.27 to 0.28) | .96 | .96 |
|  | T3-T0 | 0.14 (0.23) | 0.03 (0.21) | 0.11 (–0.22 to 0.43) | 0.10 (–0.21 to 0.41) | .53 | .69 |
| Subjective support | T1-T0 | 0.08 (0.17) | 0.01 (0.15) | 0.07 (–0.24 to 0.37) | 0.06 (–0.20 to 0.31) | .67 | .89 |
|  | T2-T0 | 0.06 (0.18) | 0.00 (0.16) | 0.05 (–0.27 to 0.38) | 0.05 (–0.23 to 0.32) | .74 | .96 |
|  | T3-T0 | 0.10 (0.27) | –0.04 (0.26) | 0.14 (–0.25 to 0.53) | 0.11 (–0.20 to 0.42) | .48 | .69 |
| Objective support | T1-T0 | –0.14 (0.15) | 0.01 (0.14) | –0.14 (–0.43 to 0.14) | –0.13 (–0.39 to 0.13) | .32 | .89 |
|  | T2-T0 | 0.01 (0.15) | 0.14 (0.14) | –0.12 (–0.39 to 0.15) | –0.12 (–0.39 to 0.15) | .38 | .96 |
|  | T3-T0 | 0.10 (0.25) | 0.17 (0.24) | –0.07 (–0.44 to 0.29) | –0.06 (–0.37 to 0.25) | .69 | .69 |
| Support utilization | T1-T0 | 0.12 (0.16) | 0.01 (0.15) | 0.11 (–0.18 to 0.41) | 0.10 (–0.16 to 0.36) | .45 | .89 |
|  | T2-T0 | 0.24 (0.16) | 0.15 (0.15) | 0.09 (–0.21 to 0.40) | 0.08 (–0.19 to 0.36) | .55 | .96 |
|  | T3-T0 | 0.20 (0.26) | –0.06 (0.25) | 0.26 (–0.13 to 0.64) | 0.21 (–0.10 to 0.52) | .19 | .69 |

## **Table S9. Between-group comparisons for scores of outcome indicators at postintervention, 1-month follow-up, and 3-month follow-up assessments in adolescents without anxiety symptoms.** T0: baseline. T1: postintervention. T2: 1-month follow-up. T3: 3-month follow-up. FDR: false discovery rate correction. ^a^ Due to baseline imbalances in objective support, support utilization, and overall social support between the intervention and control groups, comparisons of these indicators were not performed.

| **Measures** | **Time effect** | | | | **Group effect** | | | | | **Time-by-group interaction** | | | |
| --- | --- | --- | --- | --- | --- | --- | --- | --- | --- | --- | --- | --- | --- |
|  | ***F* (df)** | ***P* value** | **η^2^_p_** | **Post hoc comparisons Cohen *d* (95%CI)** | ***F* (df)** | ***P* value** | **FDR adjusted *P* value** | **η^2^_p_** | **Between-group comparisons (intervention group-control group) Cohen *d* (95%CI)** | ***F* (df)** | ***P* value** | **FDR adjusted *P* value** | **η^2^_p_** |
| **Depressive symptom severity** | *99.099 (3,531)* | *<.001* | 0.360 | *T1-T0: –1.74 (–1.99 to –1.49)*  *T2-T0: –1.59 (–1.84 to –1.33)*  *T3-T0: –1.49 (–1.75 to –1.23)* | 0.332 (1,531) | .56 | - | 0.001 | 0.05 (–0.13 to 0.23) | 1.025 (3,531) | .38 | - | 0.006 |
| **Anxiety symptom severity** | 2.207 (3,531) | .09 | 0.010 | *T1-T0: –0.23 (–0.46 to –0.00)*  *T2-T0: –0.27 (–0.50 to –0.04)*  T3-T0: –0.22 (–0.46 to 0.03) | 0.317 (1,531) | .57 | .82 | 0.001 | 0.05 (–0.13 to 0.23) | 0.032 (3,531) | .99 | .99 | <0.001 |
| **Suicidal ideation severity** | *10.968 (3,531)* | *<.001* | 0.060 | *T1-T0: –0.45 (–0.68 to –0.22)*  *T2-T0: –0.62 (–0.86 to –0.39)*  *T3-T0: –0.50 (–0.75 to –0.26)* | 0.054 (1,531) | .82 | .82 | <0.001 | 0.02 (–0.16 to 0.20) | 0.115 (3,531) | .95 | .99 | 0.001 |
| **Resilience** | 0.777 (3,531) | .51 | 0.004 | T1-T0: 0.04 (–0.19 to 0.26)  T2-T0: 0.17 (–0.06 to 0.41)  T3-T0: 0.06 (–0.19 to 0.30) | 2.771 (1,531) | .10 | .19 | 0.005 | 0.15 (–0.03 to 0.33) | 1.018 (3,531) | .38 | .61 | 0.006 |
| Emotion regulation | *9.609 (3,531)* | *<.001* | 0.050 | *T1-T0: 0.56 (0.33 to 0.79)*  *T2-T0: 0.49 (0.26 to 0.72)*  *T3-T0: 0.27 (0.03 to 0.52)* | 2.848 (1,531) | .09 | .19 | 0.005 | 0.15 (–0.03 to 0.33) | 1.034 (3,531) | .38 | .61 | 0.006 |
| Interpersonal support | *82.861 (3,531)* | *<.001* | 0.320 | T1-T0: 0.16 (–0.07 to 0.38)  T2-T0: 0.08 (–0.15 to 0.31)  *T3-T0: –1.61 (–1.87 to –1.34)* | 3.809 (1,531) | .05 | .19 | 0.007 | 0.18 (–0.00 to 0.36) | 1.167 (3,531) | .32 | .61 | 0.007 |
| Family support | 1.705 (3,531) | .16 | 0.010 | T1-T0: 0.03 (–0.20 to 0.25)  T2-T0: 0.10 (–0.13 to 0.33)  *T3-T0: 0.26 (0.02 to 0.51)* | 0.107 (1,531) | .74 | .74 | <0.001 | 0.03 (–0.15 to 0.21) | 0.090 (3,531) | .97 | .97 | 0.001 |
| Positive cognition | *89.433 (3,531)* | *<.001* | 0.340 | *T1-T0: –0.43 (–0.66 to –0.21)*  T2-T0: –0.23 (–0.46 to 0.01)  *T3-T0: 1.49 (1.23 to 1.75)* | 0.608 (1,531) | .44 | .52 | 0.001 | –0.07 (–0.25 to 0.11) | 0.972 (3,531) | .41 | .61 | 0.005 |
| Focused targeting | 2.106 (3,531) | .10 | 0.010 | *T1-T0: –0.24 (–0.46 to –0.01)*  T2-T0: 0.01 (–0.23 to 0.24)  T3-T0: –0.17 (–0.42 to 0.07) | 1.720 (1,531) | .19 | .29 | 0.003 | 0.12 (–0.06 to 0.30) | 0.730 (3,531) | .53 | .64 | 0.004 |
| **Difficulties in emotion regulation** | 2.045 (3,531) | .11 | 0.010 | T1-T0: 0.14 (–0.09 to 0.36)  T2-T0: –0.16 (–0.39 to 0.07)  T3-T0: –0.05 (–0.29 to 0.20) | 0.096 (1,531) | .76 | .88 | <0.001 | –0.03 (–0.21 to 0.15) | 0.272 (3,531) | .85 | .94 | 0.002 |
| Lack of emotional awareness | *3.773 (3,531)* | *.01* | 0.020 | *T1-T0: 0.37 (0.14 to 0.60)*  *T2-T0: 0.28 (0.05 to 0.51)*  T3-T0: 0.20 (–0.04 to 0.45) | 0.909 (1,531) | .34 | .86 | 0.002 | 0.09 (–0.09 to 0.27) | 0.128 (3,531) | .94 | .94 | 0.001 |
| Lack of emotional clarity | *4.965 (3,531)* | *.002* | 0.030 | *T1-T0: 0.36 (0.13 to 0.59)*  T2-T0: –0.02 (–0.25 to 0.21)  *T3-T0: 0.25 (0.01 to 0.50)* | 0.441 (1,531) | .51 | .86 | 0.001 | –0.06 (–0.24 to 0.12) | 0.160 (3,531) | .92 | .94 | 0.001 |
| Nonacceptance of emotional responses | 0.748 (3,531) | .52 | 0.004 | T1-T0: 0.00 (–0.22 to 0.23)  T2-T0: –0.14 (–0.38 to 0.09)  T3-T0: –0.10 (–0.35 to 0.14) | 0.258 (1,531) | .61 | .86 | <0.001 | –0.05 (–0.23 to 0.13) | 0.178 (3,531) | .91 | .94 | 0.001 |
| Impulse control difficulties | 1.080 (3,531) | .36 | 0.006 | T1-T0: 0.05 (–0.17 to 0.28)  T2-T0: –0.14 (–0.37 to 0.09)  T3-T0: –0.11 (–0.35 to 0.14) | 0.004 (1,531) | .95 | .95 | <0.001 | –0.01 (–0.19 to 0.18) | 0.300 (3,531) | .83 | .94 | 0.002 |
| Difficulties engaging in goal-directed behavior | *6.249 (3,531)* | *<.001* | 0.030 | *T1-T0: –0.33 (–0.56 to –0.11)*  *T2-T0: –0.47 (–0.71 to –0.24)*  *T3-T0: –0.38 (–0.62 to –0.13)* | 0.544 (1,531) | .46 | .86 | 0.001 | –0.07 (–0.25 to 0.11) | 0.260 (3,531) | .85 | .94 | 0.001 |
| Limited access to emotion regulation strategies | 1.074 (3,531) | .36 | 0.006 | T1-T0: 0.04 (–0.18 to 0.27)  T2-T0: –0.16 (–0.40 to 0.07)  T3-T0: –0.04 (–0.29 to 0.20) | 0.521 (1,531) | .47 | .86 | 0.001 | –0.07 (–0.25 to 0.11) | 0.451 (3,531) | .72 | .94 | 0.003 |
| **Social support** | -^a^ | - | - | - | - | - | - | - | - | - | - | - | - |
| Subjective support | 0.625 (3,531) | .60 | 0.004 | T1-T0: 0.16 (–0.07 to 0.38)  T2-T0: 0.09 (–0.14 to 0.32)  T3-T0: 0.08 (–0.16 to 0.33) | 1.332 (1,531) | .25 | - | 0.003 | 0.11 (–0.07 to 0.29) | 2.394 (3,531) | .07 | - | 0.010 |
| Objective support | - | - | - | - | - | - | - | - | - | - | - | - | - |
| Support utilization | - | - | - | - | - | - | - | - | - | - | - | - | - |

## **Table S10. Between-group comparisons for change scores in outcome indicators from baseline to postintervention, 1-month follow-up, and 3-month follow-up among adolescents without anxiety symptoms.** T0: baseline. T1: postintervention. T2: 1-month follow-up. T3: 3-month follow-up. *LSM*: least squares mean. *LSMD*: least squares mean difference. FDR: false discovery rate correction. ^a^ Due to baseline imbalances in objective support, support utilization, and overall social support between the intervention and control groups, comparisons of these indicators were not performed. At T1, T2, and T3, the intervention group included 66, 61, and 51 participants while the control group included 80, 71, and 61 participants, respectively.

| **Measures** | **Timepoints** | **Intervention group, *LSM* (*SE*)** | **Control group, *LSM* (*SE*)** | ***LSMD* (95%CI)** | **Cohen *d* (95%CI)** | ***P* value** | **FDR adjusted *P* value** |
| --- | --- | --- | --- | --- | --- | --- | --- |
| **Depressive symptom severity** | T1-T0 | –7.90 (1.04) | –7.61 (1.03) | –0.29 (–2.02 to 1.43) | –0.06 (–0.40 to 0.29) | .74 | .77 |
|  | T2-T0 | –6.31 (1.20) | –5.80 (1.21) | –0.51 (–2.50 to 1.48) | –0.09 (–0.46 to 0.27) | .61 | .84 |
|  | T3-T0 | –5.41 (1.49) | –6.46 (1.44) | 1.05 (–1.12 to 3.22) | 0.20 (–0.21 to 0.61) | .34 | .77 |
| **Anxiety symptom severity** | T1-T0 | –0.19 (0.92) | –0.42 (0.91) | 0.23 (–1.30 to 1.76) | 0.05 (–0.29 to 0.40) | .77 | .77 |
|  | T2-T0 | 0.52 (1.09) | 0.71 (1.10) | –0.19 (–2.00 to 1.62) | –0.04 (–0.41 to 0.33) | .84 | .84 |
|  | T3-T0 | 1.74 (1.36) | 1.44 (1.32) | 0.30 (–1.69 to 2.28) | 0.06 (–0.35 to 0.47) | .77 | .77 |
| **Suicidal ideation severity** | T1-T0 | –3.18 (1.64) | –3.91 (1.63) | 0.73 (–2.00 to 3.46) | 0.09 (–0.25 to 0.44) | .60 | .77 |
|  | T2-T0 | –3.53 (1.53) | –3.84 (1.54) | 0.31 (–2.22 to 2.84) | 0.05 (–0.32 to 0.41) | .81 | .84 |
|  | T3-T0 | –1.12 (2.00) | –1.93 (1.94) | 0.81 (–2.11 to 3.72) | 0.11 (–0.30 to 0.52) | .58 | .77 |
| **Resilience** | T1-T0 | –1.30 (2.41) | –1.13 (2.39) | –0.17 (–4.17 to 3.84) | –0.01 (–0.36 to 0.33) | .93 | .97 |
|  | T2-T0 | 4.61 (3.64) | 0.07 (3.66) | 4.55 (–1.48 to 10.60) | 0.28 (–0.09 to 0.65) | .14 | .49 |
|  | T3-T0 | 0.63 (4.09) | –2.08 (3.95) | 2.71 (–3.23 to 8.66) | 0.19 (–0.22 to 0.59) | .37 | .73 |
| Emotion regulation | T1-T0 | 2.81 (1.07) | 2.10 (1.06) | 0.71 (–1.06 to 2.48) | 0.14 (–0.21 to 0.48) | .43 | .97 |
|  | T2-T0 | 1.92 (1.29) | 0.86 (1.29) | 1.06 (–1.07 to 3.18) | 0.18 (–0.19 to 0.55) | .33 | .49 |
|  | T3-T0 | 1.35 (1.29) | 0.30 (1.25) | 1.04 (–0.83 to 2.92) | 0.23 (–0.18 to 0.64) | .27 | .73 |
| Interpersonal support | T1-T0 | 0.12 (0.89) | 0.14 (0.88) | –0.03 (–1.50 to 1.45) | –0.01 (–0.35 to 0.34) | .97 | .97 |
|  | T2-T0 | 0.38 (1.08) | –0.63 (1.08) | 1.01 (–0.77 to 2.79) | 0.21 (–0.16 to 0.58) | .26 | .49 |
|  | T3-T0 | –6.74 (1.54) | –7.12 (1.49) | 0.38 (–1.87 to 2.62) | 0.07 (–0.34 to 0.48) | .74 | .94 |
| Family support | T1-T0 | –1.27 (0.84) | –0.64 (0.84) | –0.63 (–2.04 to 0.77) | –0.16 (–0.50 to 0.19) | .38 | .97 |
|  | T2-T0 | 0.64 (1.19) | 0.18 (1.19) | 0.47 (–1.50 to 2.43) | 0.09 (–0.28 to 0.46) | .64 | .64 |
|  | T3-T0 | 0.53 (1.71) | 0.43 (1.65) | 0.09 (–2.39 to 2.58) | 0.02 (–0.39 to 0.42) | .94 | .94 |
| Positive cognition | T1-T0 | –1.73 (0.84) | –1.60 (0.84) | –0.14 (–1.54 to 1.27) | –0.03 (–0.38 to 0.31) | .85 | .97 |
|  | T2-T0 | –0.13 (1.06) | –0.78 (1.07) | 0.65 (–1.11 to 2.41) | 0.14 (–0.23 to 0.51) | .46 | .56 |
|  | T3-T0 | 4.54 (1.43) | 4.72 (1.38) | –0.18 (–2.25 to 1.90) | –0.04 (–0.44 to 0.37) | .86 | .94 |
| Focused targeting | T1-T0 | –1.22 (1.12) | –1.14 (1.11) | –0.08 (–1.95 to 1.78) | –0.02 (–0.36 to 0.33) | .93 | .97 |
|  | T2-T0 | 1.80 (1.23) | 0.44 (1.24) | 1.36 (–0.68 to 3.39) | 0.25 (–0.12 to 0.62) | .19 | .49 |
|  | T3-T0 | 0.96 (1.69) | –0.42 (1.64) | 1.38 (–1.08 to 3.84) | 0.23 (–0.18 to 0.64) | .27 | .73 |
| **Difficulties in emotion regulation** | T1-T0 | 2.92 (4.07) | –1.19 (4.05) | 4.11 (–2.66 to 10.90) | 0.21 (–0.14 to 0.56) | .23 | .65 |
|  | T2-T0 | –0.15 (4.57) | –0.75 (4.59) | 0.60 (–6.97 to 8.16) | 0.03 (–0.34 to 0.40) | .88 | .96 |
|  | T3-T0 | 9.86 (6.42) | 8.95 (6.21) | 0.91 (–8.44 to 10.30) | 0.04 (–0.37 to 0.45) | .85 | .96 |
| Lack of emotional awareness | T1-T0 | 1.41 (1.44) | 1.06 (1.43) | 0.36 (–2.05 to 2.76) | 0.05 (–0.29 to 0.40) | .77 | .78 |
|  | T2-T0 | 0.50 (1.52) | 0.69 (1.52) | –0.19 (–2.70 to 2.32) | –0.03 (–0.40 to 0.34) | .88 | .96 |
|  | T3-T0 | 0.45 (1.96) | 0.53 (1.89) | –0.08 (–2.93 to 2.77) | –0.01 (–0.42 to 0.40) | .96 | .96 |
| Lack of emotional clarity | T1-T0 | 0.69 (0.70) | 0.85 (0.69) | –0.16 (–1.32 to 1.00) | –0.05 (–0.39 to 0.30) | .78 | .78 |
|  | T2-T0 | –0.47 (0.80) | –0.23 (0.80) | –0.24 (–1.56 to 1.09) | –0.07 (–0.43 to 0.30) | .72 | .96 |
|  | T3-T0 | 2.32 (1.05) | 2.73 (1.02) | –0.41 (–1.95 to 1.12) | –0.11 (–0.52 to 0.30) | .60 | .96 |
| Nonacceptance of emotional responses | T1-T0 | 0.47 (1.48) | –0.59 (1.47) | 1.06 (–1.41 to 3.53) | 0.15 (–0.20 to 0.49) | .40 | .65 |
|  | T2-T0 | 0.10 (1.69) | 0.17 (1.70) | –0.07 (–2.87 to 2.74) | –0.01 (–0.38 to 0.36) | .96 | .96 |
|  | T3-T0 | 2.45 (2.08) | 2.22 (2.02) | 0.23 (–2.81 to 3.26) | 0.03 (–0.38 to 0.44) | .88 | .96 |
| Impulse control difficulties | T1-T0 | 1.10 (1.20) | 0.36 (1.19) | 0.74 (–1.26 to 2.74) | 0.13 (–0.22 to 0.47) | .47 | .65 |
|  | T2-T0 | 1.00 (1.27) | –0.36 (1.28) | 1.36 (–0.74 to 3.46) | 0.24 (–0.13 to 0.61) | .20 | .96 |
|  | T3-T0 | 2.46 (1.64) | 1.84 (1.59) | 0.62 (–1.77 to 3.01) | 0.11 (–0.30 to 0.51) | .61 | .96 |
| Difficulties engaging in goal-directed behavior | T1-T0 | –1.33 (0.93) | –2.17 (0.92) | 0.83 (–0.71 to 2.38) | 0.19 (–0.16 to 0.53) | .29 | .65 |
|  | T2-T0 | –0.92 (1.03) | –1.29 (1.04) | 0.37 (–1.34 to 2.08) | 0.08 (–0.29 to 0.45) | .67 | .96 |
|  | T3-T0 | 0.11 (1.35) | –0.66 (1.31) | 0.77 (–1.20 to 2.74) | 0.16 (–0.25 to 0.57) | .44 | .96 |
| Limited access to emotion regulation strategies | T1-T0 | 0.58 (1.50) | –0.70 (1.49) | 1.28 (–1.22 to 3.78) | 0.18 (–0.17 to 0.52) | .31 | .65 |
|  | T2-T0 | –0.36 (1.64) | 0.28 (1.65) | –0.64 (–3.36 to 2.07) | –0.09 (–0.46 to 0.28) | .64 | .96 |
|  | T3-T0 | 2.07 (2.34) | 2.29 (2.26) | –0.22 (–3.62 to 3.19) | –0.03 (–0.43 to 0.38) | .90 | .96 |
| **Social support** | T1-T0 | -^a^ | - | - | - | - | - |
|  | T2-T0 | - | - | - | - | - | - |
|  | T3-T0 | - | - | - | - | - | - |
| Subjective support | T1-T0 | 0.28 (0.16) | 0.26 (0.16) | 0.03 (–0.25 to 0.30) | 0.03 (–0.31 to 0.38) | .86 | - |
|  | T2-T0 | 0.16 (0.23) | –0.20 (0.24) | 0.35 (–0.03 to 0.74) | 0.34 (–0.03 to 0.70) | .07 | - |
|  | T3-T0 | –0.08 (0.30) | 0.06 (0.29) | –0.14 (–0.57 to 0.29) | –0.13 (–0.54 to 0.28) | .53 | - |
| Objective support | T1-T0 | - | - | - | - | - | - |
|  | T2-T0 | - | - | - | - | - | - |
|  | T3-T0 | - | - | - | - | - | - |
| Support utilization | T1-T0 | - | - | - | - | - | - |
|  | T2-T0 | - | - | - | - | - | - |
|  | T3-T0 | - | - | - | - | - | - |

## **Sensitivity Analysis Results**

The full sample had a mean baseline Patient Health Questionnaire-8 (PHQ-8) score of 14.43 (SD 3.02), with scores ranging from 10 to 24. Specially, 8.9% (39/436) of participants had PHQ-8 scores between 10 and 11, which were exactly at or marginally above the study’s inclusion criterion (PHQ-8 ≥10). The corresponding proportions in the subgroups with and without anxiety symptoms were 6.2% (17/275) and 13.7% (22/161), respectively.

We conducted sensitivity analyses to address the potential impact of regression to the mean and measurement noise around the diagnostic threshold. Participants with baseline PHQ-8 scores of 10 to 11 were excluded from the analysis, as these individuals were most susceptible to spurious remission driven by random measurement error. After this exclusion, several positive findings remained statistically significant, confirming the robustness of our main results related to group differences in outcomes.

A total of 8.9% (39/436) of participants in the full sample were excluded. The difference in changes in the interpersonal support dimension of resilience from T0 to T1 remained significant (intervention group: mean change 1.97, SD 0.59; control group: mean change 0.62, SD 0.57; *LSMD*=1.35, 95%CI 0.35 to 2.35; Cohen *d*=0.28, 95%CI 0.07 to 0.49, *P*=.008).

Among adolescents with anxiety symptoms, 6.2% (17/275) were excluded. Significant group differences still existed for changes in the interpersonal support dimension of resilience from T0 to T1 (intervention group: mean change 2.54, SD 0.74; control group: mean change 0.74, SD 0.69; *LSMD*=1.79, 95%CI 0.50 to 3.08; Cohen *d*=0.37, 95%CI 0.10 to 0.64, *P*=.007) and depressive symptom remission rate at T1 (adjusted OR=1.35, 95%CI 1.01 to 1.81; *P*=.04; NNT=8). The mediation analysis also yielded a significant indirect association across the sequence: group → changes in interpersonal support from T0 to T1 → depressive symptom remission at T1 (β=0.07, 95%CI 0.01 to 0.13, *P*=.02), and the proportion mediated was 49.2% (95%CI 3.2% to 215.8%).
